# Supplementary material for: Anaerobic methanotrophic communities thrive in deep submarine permafrost
Source: Sci Rep. 2018 Jan 22;8:1291. doi: 10.1038/s41598-018-19505-9 (PMC5778128; doi:10.1038/s41598-018-19505-9)
Supplement: Supplementary file 1 — Supplementary Information [file 41598_2018_19505_MOESM1_ESM.doc]

Title

Anaerobic methanotrophic communities thrive in deep submarine permafrost

**Authors**

Matthias Winkel^1,*^, Julia Mitzscherling^1^, Pier P. Overduin^2^, Fabian Horn^1^, Maria Winterfeld^3^, Ruud Rijkers^1^, Mikhail N. Grigoriev^4^, Christian Knoblauch^5^, Kai Mangelsdorf^6^, Dirk Wagner^1^, and Susanne Liebner^1^

**Affiliations**

^1^GFZ German Research Centre for Geosciences, Helmholtz Centre Potsdam, Section 5.3 Geomicrobiology, 14473 Potsdam, Germany

^2^Alfred Wegener Institute, Helmholtz Centre for Polar and Marine Research, Periglacial Research, 14473 Potsdam, Germany

^3^Alfred Wegener Institute, Helmholtz Centre for Polar and Marine Research, Marine Geochemistry, 27570 Bremerhaven, Germany

^4^Mel’nikov Permafrost Institute, SB RAS, Yakutsk, 677010 Russia

^5^Institute of Soil Science, Universität Hamburg, 20146 Hamburg, Germany

^6^GFZ German Research Centre for Geosciences, Helmholtz Centre Potsdam, Section 3.2 Organic Geochemistry, 14473 Potsdam, Germany

*Corresponding author

E-mail: mwinkel@gfz-potsdam.de

Address: Telegrafenberg, building F, room 357, 14473 Potsdam, Germany

Supplementary Materials

**Methods**

**Illumina Sequence analysis**

The quality of the sequences was checked using the fastqc tool. (FastQC A Quality Control tool for High Throughput Sequence Data http://www.bioinformatics.babraham.ac.uk/projects/fastqc/ by S. Andrews). Sequence raw reads were demultiplexed (Table S3) and barcodes were removed with the CutAdapt tool [trim-n; e 0.1; only consider exact barcodes for mapping]^1^. The subsequent steps included merging of reads using overlapping sequence regions using PEAR [Q 25; p 0.0001; v 20]^2^, standardizing the nucleotide sequence orientation, and trimming and filtering of low quality sequences using Trimmomatic [SE; LEADING Q25; TRAILING Q25; SLIDINGWINDOW 5:25; MINLEN 200]^3^. After trimming we performed a chimera check with ChimeraSlayer^4^ and removed sequences from the dataset. Subsequently sequences were clustered into operational taxonomic units (OTU) by usearch v6.1^5^ at a nucleotide cutoff level of 97% similarity and taxonomically assigned employing the GreenGenes database 13.05^6^ using the pick_open_reference approach of the QIIME pipeline^7^. The OTU table was filtered for singletons, chloroplasts, mitochondrial and bacterial sequences. Older taxonomic assignments for archaea were corrected manually after^8–10^ e.g. Micellaneous Crenarchaeal Group (MCG) was renamed to *Bathyarchaeota*. OTUs with relative abundance lower than 0.1% for the individual libraries were not analyzed. Statistics of the sequence pipeline plus representative taxa are listed in Table S3. The full diversity of all archaeal sequences are listed in the Table S1.

**Analysis of 454 functional sequences**

Raw sequences were processed by the mothur software package (v.1.34.4)^11^ by a modified standard operating procedure (SOP). Sequences were quality filtered by removing sequences with barcode or primer errors, homopolymers longer than 8, and or average quality scores less than 25. Afterwards unique sequences were aligned against an in-house reference *mcrA* database^12^ on a nulceotide alignment. A chimera check using the processed sequences as own reference let to a detection of up to 20% chimeras but manual reviewing against the non-redundant RefSeq NCBI database^13^ showed no chimeric blast hits and therefore sequences were not excluded from the analysis. Sequences were clustered using the furthest neighbor method and OTUs were assigned at a cutoff value of 0.16^13^. OUT sequences were checked for translation errors in the ARB environment^14^, aligned and taxonomical classified by a manually curated ARB database on amino acid level with clustering at 85.7% and 75.4% amino acid similarity after^15^.

**CARD-FISH**

PFA fixed samples were washed twice in 1× phosphate-buffered saline (PBS) and stored in PBS/ethanol (1:1, vol/vol) at −20°C. Sediments were filtered on polycarbonate membranes (0.2 μm pore size; 25 mm diameter; Millipore) and CARD-FISH on all samples was performed according to Ishii and colleagues (2004)^16^ using an fluorescin-labelled tyramide. Archaeal cells were permeabilized using proteinase K according to Teira and colleagues (2004)^17^. Filter sections were embedded with a mix of Citifluor : VECTASHIELD [4:1] (VECTASHIELD® Mounting Medium H-1000, Vector Laboratories and Citifluor) containing 4′,6′-diamidino-2-phenylindole at a final concentration of 1 μg ml^−1^. Preparations were examined under a fluorescent microscope Leica DM 2000 with camera DFC 420C and filter systemFI/RH (Leica). Image stacks were merged with the ‘Picolay’ software (www.picolay.de) to get focused images of the consortia.

**Microbial lipid biomarker extraction**

Sediment samples were freeze-dried and ground. Between 4.3-9.5 g of ground sediment was mixed with 50 ml of the extraction mixture composed of methanol/dichloromethane/ammonium acetate buffer (2:1:0.8, pH 7.6) and extracted by ultra-sonication for 15 min. Afterwards, samples were centrifuged at 2500 rpm for 10 min. The solvent was transferred to a separation funnel. As internal standard, 50 μg of 1-myristyl-(D27)-2-hydroxy-*sn*-glycerol-3-phosphocholine was added. The remaining sediment was extracted another 2 times with 50 ml of the extraction mixture. For phase separation, the combined three supernatants were adjusted with dichlormethane and water to achieve a ratio of 1:1:0.9. Afterwards the organic phase was transferred into a TurboVap® 500 system (Biotage) and the remaining water phase was re-extracted 2 times with 10 ml dichloromethane. The combined organic phases were evaporated and finally dried with a gentle stream of nitrogen. Subsequently, the obtained sediment extract was separated into fractions of different polarity (low polar lipids, free FAs, glycolipids, and phospholipid PLs) by using different columns. The first column was a pure silica column (1 g silica gel 63–200 μm) combined with a downstream Florisil® column (1g magnesium silica gel 150-250 µm). According to the method described by Zink and Mangelsdorf^18^, the low polar fraction was eluted with 20 mL of chloroform, the free FAs with 50 mL of methyl formate blended with 12.5 μL of glacial acetic acid and the glycolipid fraction with 20 mL of acetone. After removal of the Florisil® column, the PLs were eluted with 25 mL of methanol from the silica column. To improve the recovery of PLs, the silica column was rinsed with 25 mL of a methanol/water mixture (60:40) and the extract was captured in a separation funnel. Dichloromethane and water were added for phase separation (methanol/dichloromethane/water, 1:1:0.9); the organic phase was removed, and the water phase was re-extracted 2 times with dichloromethane. Finally, the organic phases were combined with the PL fraction and all fractions were evaporated to dryness and stored at -20°C until analysis.

**Detection of Phospholipid Fatty Acids (PLFA), Glycerol Dialkyl Glycerol Tetraethers (GDGT) and Archaeol**

For the detection of glycerol dialkyl glycerol tetraethers (GDGT) and archaeol, the low polar lipid fraction was dissolved in 250 µl dichloromethane/methanol (99:1), and a 40-fold excess of *n*-hexane (10 ml) was added to precipitate asphaltenes. Asphaltenes were removed via filtration over sodium sulfate. The extract was separated into an aliphatic/alicyclic and an aromatic hydrocarbon fraction as well as into a polar fraction containing nitrogen, sulphur, and oxygen (NSO) bearing compounds using a medium-pressure liquid chromatography (MPLC). Subsequently, all fractions were evaporated to dryness and stored at -20°C until analysis and directly used for measurements.

Half of the PL fraction was used for PLFA analysis by a mild alkaline hydrolysis via ester cleavage^19^. For identification of the resulting fatty acids, a gas chromatograph (Trace GC Ultra, Thermo Electron Corporation) equipped with a cold injection system (Thermo Electron Corporation) and a 50 m × 0.22 mm × 0.25 μm BPX5 (SGE) column coupled to a DSQ Mass Spectrometer (Thermo Finnigan Quadrupole MS, Thermo Electron Corporation) were used. The gas chromatograph were run in splitless mode with the following setup: initial oven temperature 50 °C (1 min isothermal), heating rate 3 °C/min to 310 °C (held for 30 min) and injection temperature from 50 to 300 °C at a rate of 10 °C/s. Helium was used as carrier gas with a constant flow of 1 ml min^-1^. The mass spectrometer operated in the electron impact mode at 70 eV. Full-scan mass spectra were recorded from m/z 50–650 at a scan rate of 1.5 scans s^-1^. The measurement of GDGTs were performed with a Shimadzu LC20AD HPLC instrument coupled to a Finnigan TSQ 7000 triple quadrupole MS with an atmospheric pressure chemical ionization (APCI) interface following a method described by Schouten et al.^20^.

**Results**

In the SMTZ, the concentrations of archaeal lipids (archaeol and isoprenoid glycerol dialkyl glycerol tetraether, iGDGTs) and bacterial lipids (branched GDGTs and phospholipid fatty acids, PLFAs) were in the lower range typically observed in subsurface sediments (ng g^-1^ sediment for ether lipids and µg g^-1^ for PLFAs)^21^. These reflect globally observed cell abundances in the subsurface that can be very low beneath 10 mbsf (10^3^ to 10^8^ per cm^3^)^22^. Unfortunately, concentrations were too low to determine δ^13^C isotope values of specific lipids. Nevertheless, the ratio of archaeal (archaeol + isoprenoid GDGTs) ether lipids to bacterial branched GDGT showed almost exclusively archaeal lipids in the SMTZ, whereas ratios in layers above the SMTZ showed significantly lower ratios (Fig. S9). Moreover, we detected some PLFAs such as C_16:1ω7c_, C_18:1ω9_ and C_18:1ω7c_ in significant proportions (2.64 to 10.7%, Table S7) that have been shown to be related to bacteria involved in or influenced by AOM processes in coastal wetlands and grasslands^23,24^. However, the concentrations of these specific PLFAs were also too low to determine their carbon isotope signature.

**Discussion**

The absence of PLFAs such C_16:1ω5_, cyC_17:0ω5,6_ and C_17:1ω6c_ (Table S7) known to represent *Desulfosarcina/Desulfococcus* SRB in AOM consortia of methane seep sediments^25^ can be explained by a low biomass typical for such deep habitats. Alternatively, the seep-associated *Desulfobacterium anilini*-group may have a different lipid biomarker pattern or unknown bacterial partner are associated with ANMEs at submarine permafrost thaw fronts.

The presence and partly enrichment of PLFAs C_16:1ω7c_, C_18:1ω9_ and C_18:1ω7c_ (Table S7), which were shown to be related to AOM in peatland environments^23,24^, likely reflect other SRB communities responsible in this untypical SMTZ in deep submarine permafrost.

The ether-lipid ratios of the SMTZ were untypical for permafrost (Fig. S9), since analyses of terrestrial and lacustrine permafrost showed most of the time higher concentrations for branched GDGTs over archaeol and isoprenoid GDGTs in the same sediment horizons^21,26^.

**Supplementary Figures**

**Fig. S1.** Phylogenetic affiliation of ANME sequences based on 16S rRNA. Sequences in bold represent OTUs from the Illumina sequencing and clone library sequences. *Methanopyrus kandleri* was used as outgroup. The scale bar represents 10 percent sequence divergence.

Figure S1 is provided as a separate PDF file.

**Fig. S2.** Phylogenetic affiliation of archaeal sequences based on 16S rRNA. Sequences in bold represent OTUs from the Illumina sequencing and clone library sequences. *Escherichia coli* was used as outgroup. The scale bar represents 10 percent sequence divergence.

Figure S2 is provided as a separate PDF file.

**
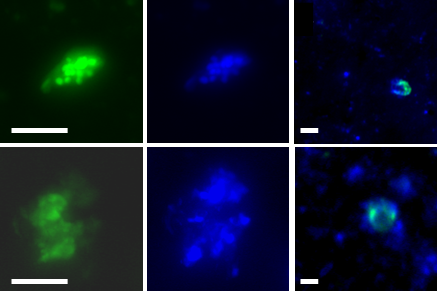
**

**Fig. S3.** Micrographs of ANME-2a consortia at the SMTZ of submarine permafrost core BK2. Anaerobic methanotrophic cells of the 2a clade were detected by CARD-FISH (probe: ANME-2a-647 - green) first column and corresponding counterstaining with DAPI (blue) second column. The third column shows overlays of ANME-2a (green) and DAPI (blue). The scale bar represents 5 µm. The first two image columns were created by Picolay (http://www.picolay.de/, © Herbiert Cypionka)


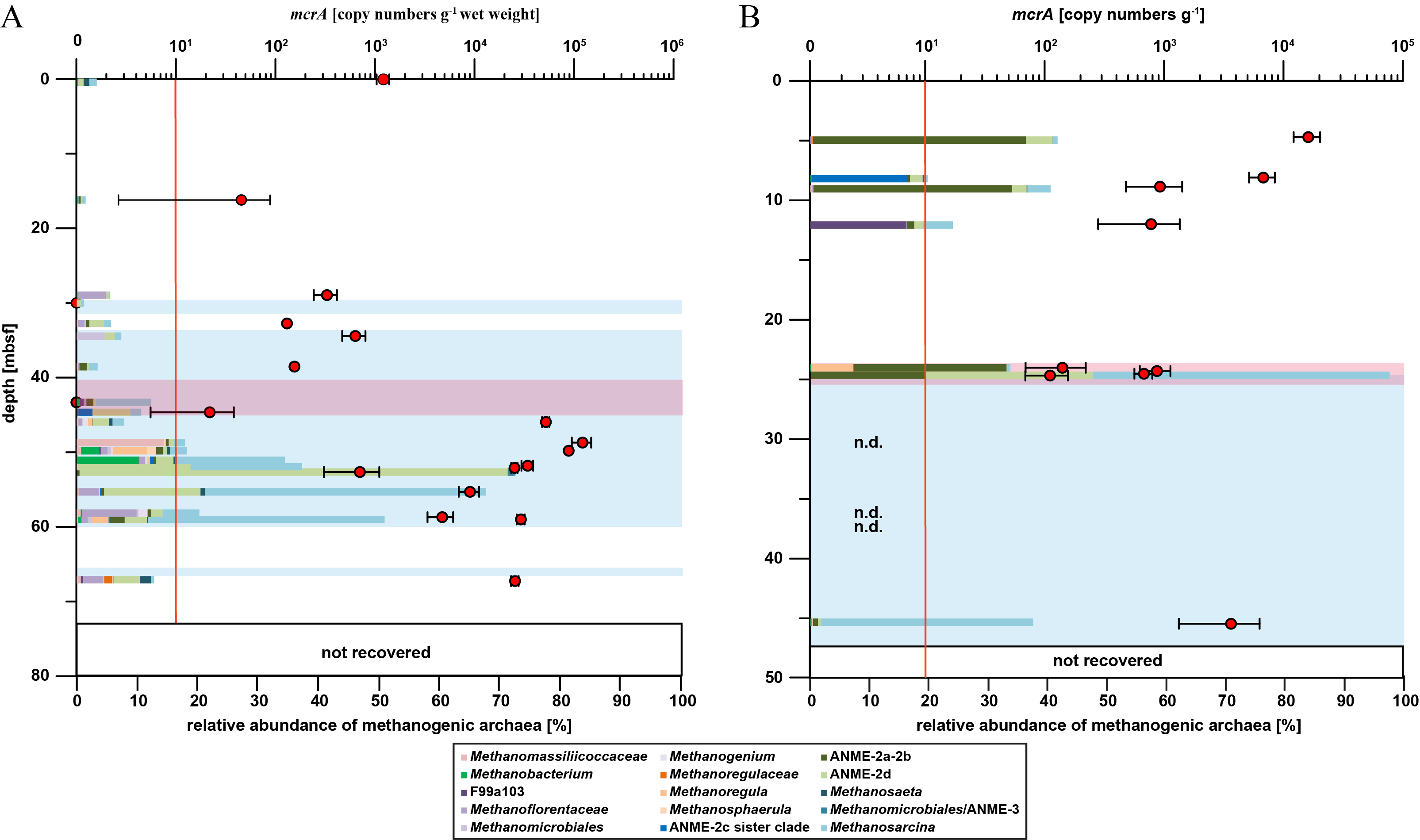


**Fig. S4.** Methanogenic/ANME communities of core C2 (A) and BK2 (B). Red circles show *mcrA* copy numbers per gram sediment wet weight as detected by quantitative PCR. The red line represents the detection limit of the qPCR related to the lowest standard. Bar charts show relative abundance of archaeal 16S rRNA sequences related to known methanogenic and methanotrophic archaea. The blue shaded area represents ice-bonded permafrost, the red area the SMTZ. All uncolored areas of the plots correspond to unfrozen submarine permafrost and marine sediments. n. d. - no detection.


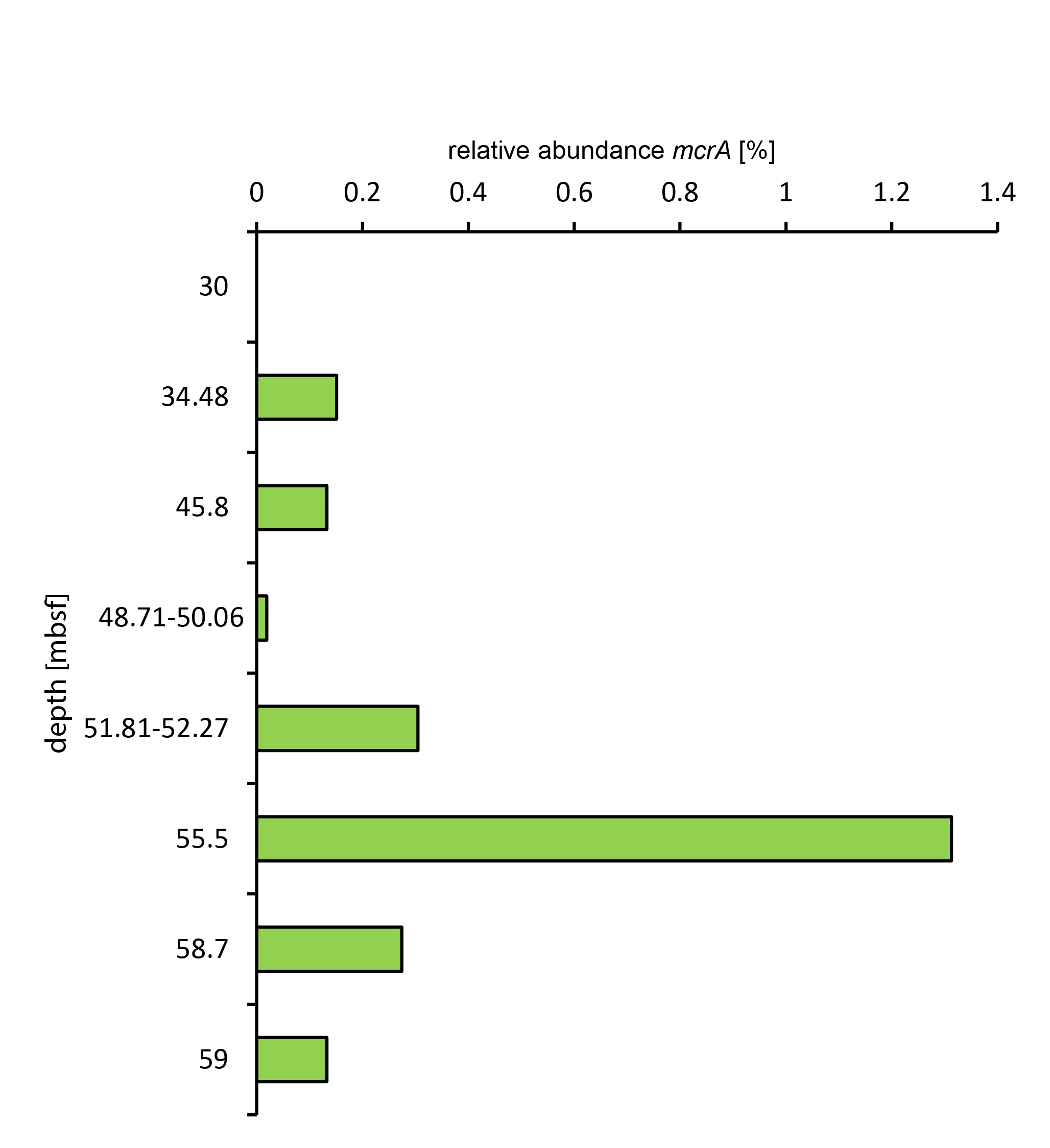


**Fig. S5.** Relative abundance of ANME-2d-related *mcrA* sequences in submarine core C2. *McrA* sequences are based on 454 sequencing.


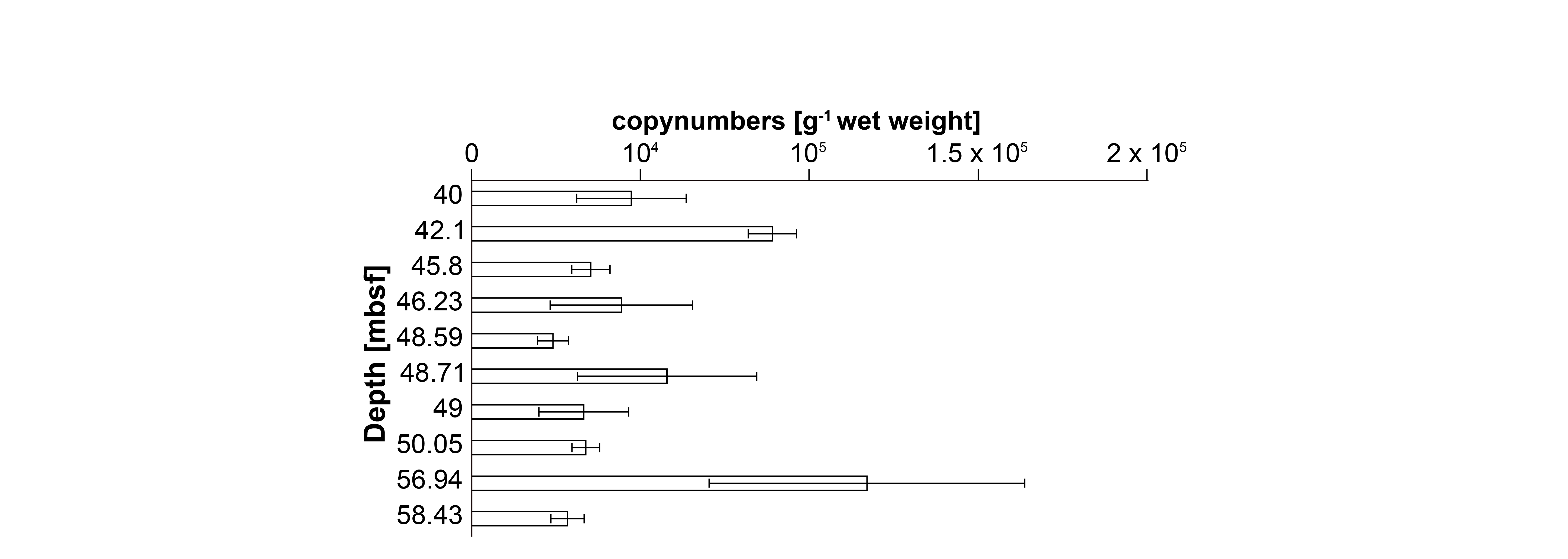


**Fig. S6.** ANME-2d specific *mcrA* copy numbers of core C2. The sampling scheme along the depth is not identical for the *mcrA* quantification and the next-generation sequencing..


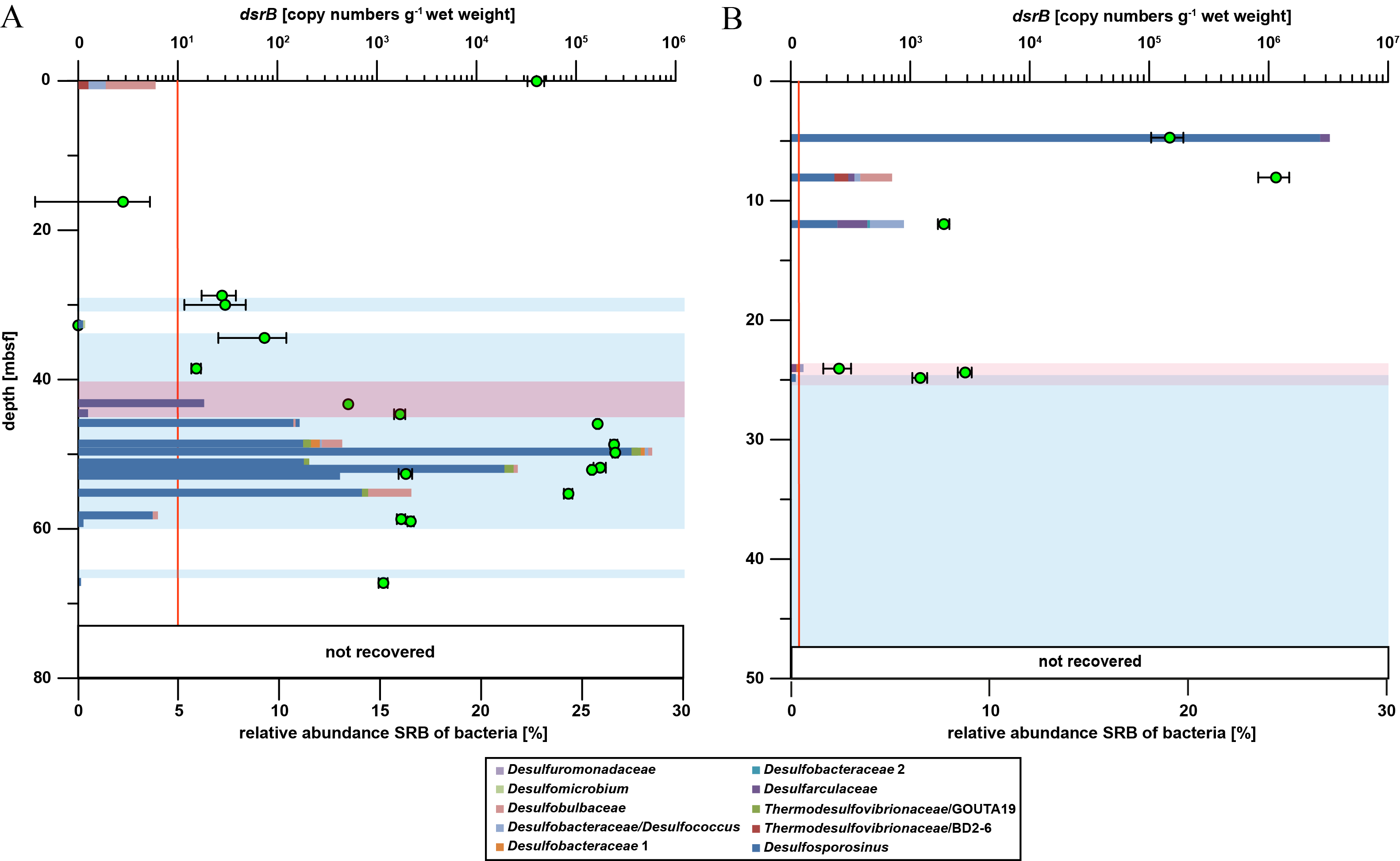


**Fig. S7.** Sulfate-reducing bacteria of core C2 (A) and BK2 (B). Green circles show *dsrB* copy numbers per gram sediment wet weight as detected by quantitative PCR. The red line represents the detection limit of the qPCR related to the lowest standard. Bar charts show relative abundance of bacterial 16S rRNA sequences related to known sulfate reducers. The blue shaded areas represent ice-bonded permafrost and the red area the SMTZ. All uncolored areas of the plots correspond to unfrozen (ice-free) submarine permafrost and marine sediments.

**
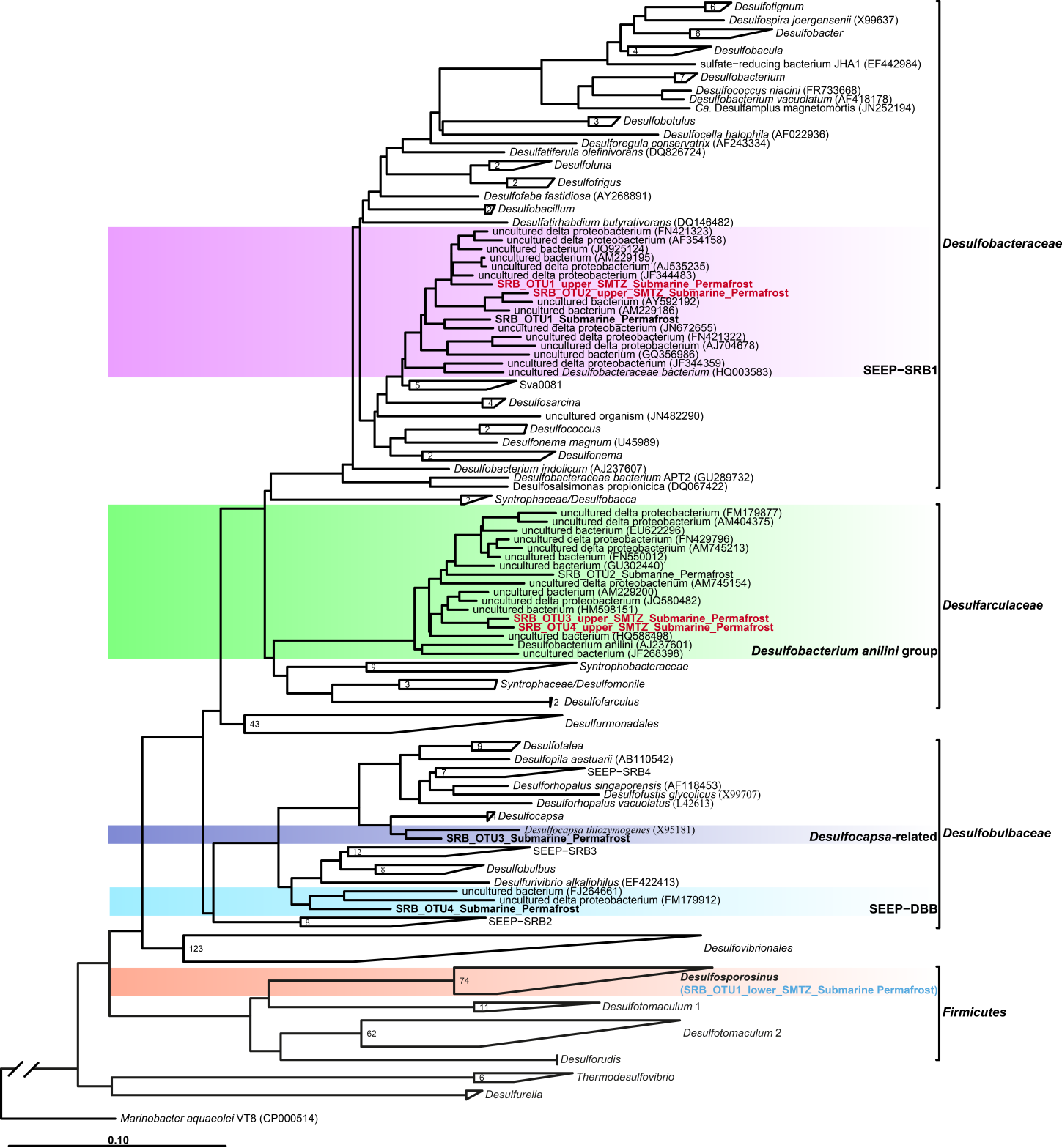
**

**Fig. S8. Phylogenetic reconstruction of 16S rRNA sequences related to sulfate-reducing bacteria.** Operational taxonomic units belonging to the upper part of the SMTZ are shown in bold-red, of the lower part in bold-blue and all other submarine permafrost in bold-black. The scale bar represents 10% sequence divergence. The colored boxes highlights cluster of SRB in submarine permafrost.


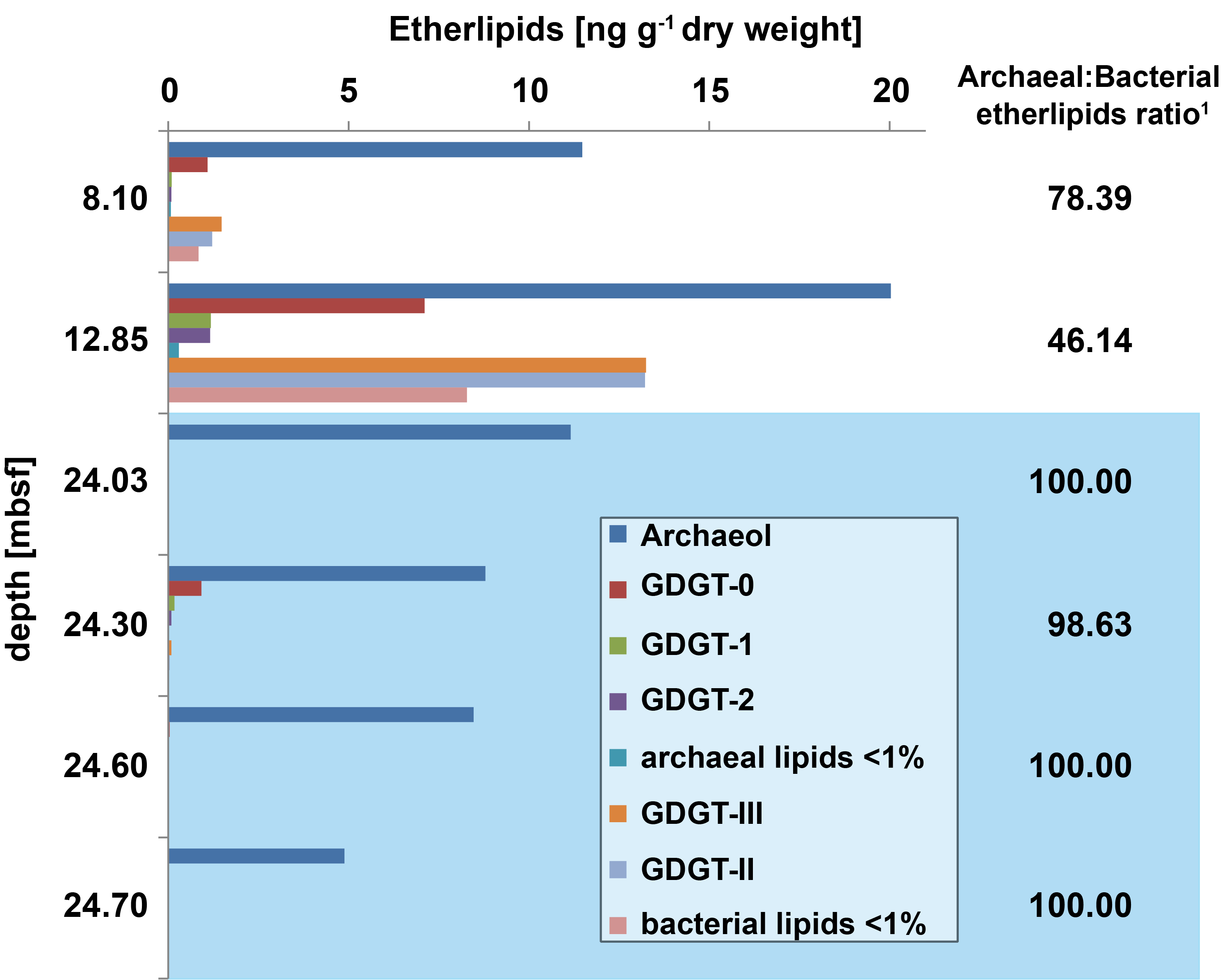


**Fig. S9.** Concentrations of archaeal and bacterial ether-lipids in submarine permafrost core BK2. The upper two samples represent marine-influenced submarine permafrost layers. while the lower four samples represent SMTZ layers (light blure rectangle). Ratios of total archaeal vs. total bacterial ether-lipids for each sediment horizon are shown on the right side.

^1^ Calculation of ratio (total archaeal ether lipids/(total archaeal + bacterial ether lipids)*100), whereas total archaeal ether lipids comprise archaeol and isoprenoid glycerol dialkyl glycerol tetraethers with non to 2 cyclopentyl rings (GDGT-0 to 2). Bacterial ether lipids represent branched GDGT-II and –III, for structures see^27^.


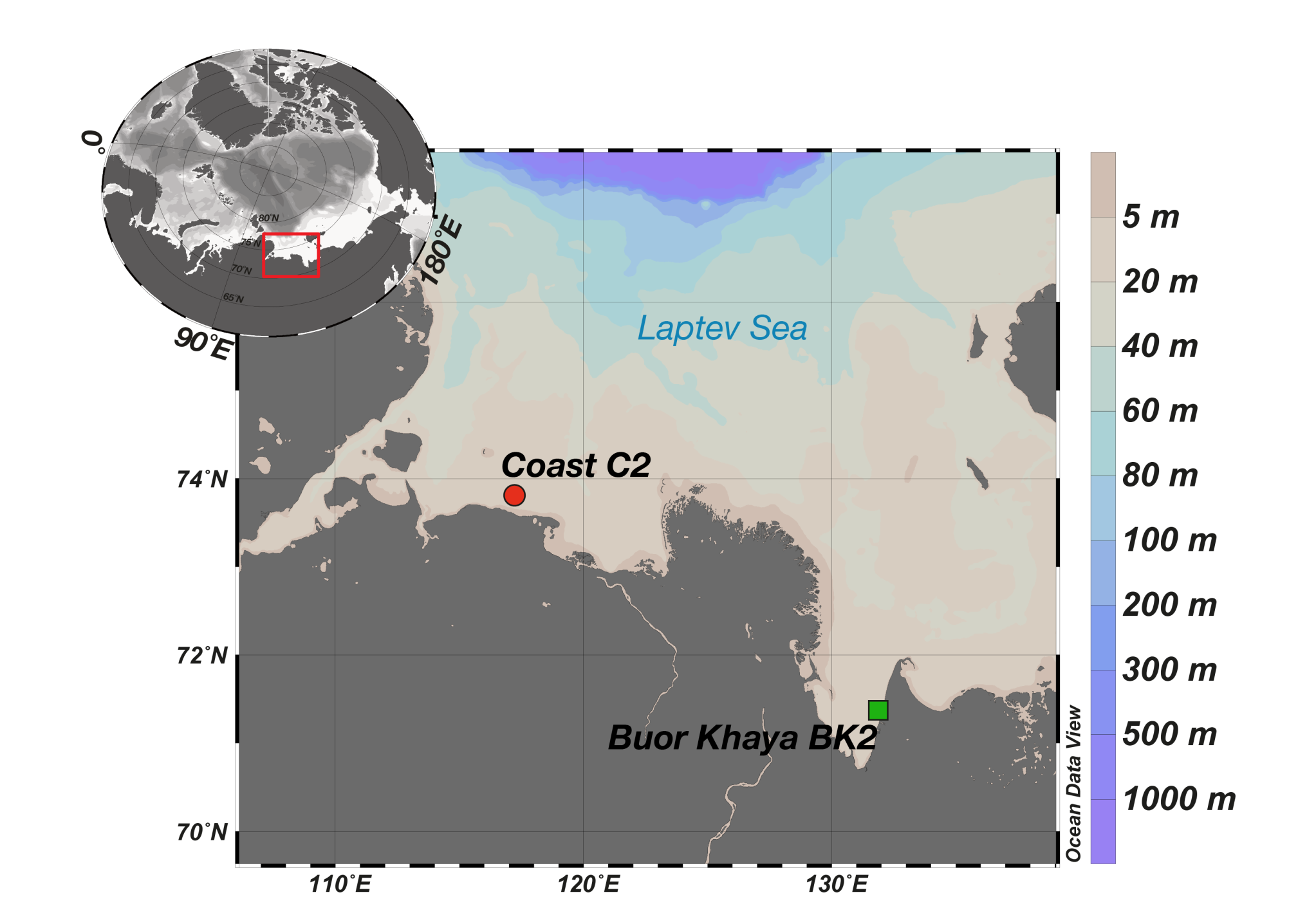


**Fig. S10.** Geographical location of sampling sites. Globe in the upper left corner shows the arctic region with the red rectangle showing the large map. Large map shows the geographical locations of the submarine permafrost cores C2 (red dot) and BK2 (green square) with ocean depth in meter according to the color code on the right hand side. The map was produced with the free software Ocean Data View version 4.7.8 (Schlitzer, R., Ocean Data View, [odv.awi.de](https://odv.awi.de/), 2017).

**Supplementary Tables**

**Table S1.** Relative abundance of archaeal sequences in both submarine permafrost cores C2 and BK2.

The table is provided as a PDF file.

**Table S2.** Characteristics of submarine permafrost cores

|  | **Mamontov Klyk (C2)** | **Buor Khaya (BK2)** |
| --- | --- | --- |
| **number of molecular samples** | 19 (Illumina and 454) and 8 (*mcrA* ANME-2d) | 10 (for DNA), 6 for (lipid analysis) |
| **estimated time of inundation [years]** | ~560 | ~2,500 |
| **maximum depth [m bsl]** | 77 | 51.7 |
| **water depth [m]** | 6 | 4.3 |
| **sea ice cover [m]** | 1.35 | 2.09 |
| **permafrost boundary [mbsf]** | 29.5 | 24.45 |
| **mean core temperature [°C]** | -1.2±0.2 | -0.5±0.4 |
| **distance to coast [km]** | 11.5 | 0.8 |
| **latitude** | 73° 36' 21.5'' N | 71° 25' 20.3'' N |
| **longitude** | 117° 10' 01.1'' E | 132° 05' 05.3'' E |

**Table S3.** Statistics of sequence analysis pipeline and representative archaeal taxa.

| **Sample site** | **Depth [m]** | **Raw reads** | **Quality reads** | **Observed OTU_0.03_** | **Inverse Simpson** | **Shannon** | **Most frequent class*** | **Most frequent order*** |
| --- | --- | --- | --- | --- | --- | --- | --- | --- |
| **Mamontov Klyk** | 0.50 | 266,820 | 245,919 | 85 | 11.94 | 3.24 | unknown *Thaumarchaeota* | *Nitrosopumilus* |
|  | 18.00 | 97,307 | 92,379 | 44 | 4.26 | 1.97 | unknown *Bathyarchaeota* | MCG-6 |
|  | 29.00 | 159,157 | 146,155 | 63 | 2.72 | 1.97 | *Diaforarchaea* | DSPEG-I |
|  | 30.00 | 199,727 | 189,569 | 38 | 2.00 | 1.49 | *Diaforarchaea* | DSPEG-I |
|  | 33.00 | 72,724 | 68,147 | 52 | 2.97 | 1.99 | *Diaforarchaea* | DSPEG-I |
|  | 34.43 | 115,192 | 105,217 | 44 | 2.45 | 1.77 | *Diaforarchaea* | DSPEG-I |
|  | 38.53 | 170,804 | 161,167 | 45 | 1.82 | 1.25 | *Diaforarchaea* | DSPEG-I |
|  | 43.31 | 213,945 | 141,103 | 51 | 3.27 | 2.07 | *Diaforarchaea* | DSPEG-I |
|  | 44.65 | 182,186 | 162,813 | 59 | 2.38 | 1.84 | *Diaforarchaea* | DSPEG-I |
|  | 45.80 | 286,328 | 258,712 | 64 | 4.63 | 2.35 | *Nitrosospheria* | *Nitrososphaerales* |
|  | 48.71 | 130,327 | 126,630 | 25 | 3.29 | 1.58 | unknown *Bathyarchaeota* | MCG-6 |
|  | 50.06 | 132,523 | 83,306 | 84 | 9.21 | 3.12 | unknown *Bathyarchaeota* | MCG-6 |
|  | 51.81 | 151,919 | 125,190 | 84 | 8.21 | 2.79 | unknown *Bathyarchaeota* | MCG-6 |
|  | 52.27 | 325,097 | 307,942 | 53 | 4.53 | 2.32 | unknown *Bathyarchaeota* | MCG-6 |
|  | 52.69 | 269,218 | 258,031 | 27 | 4.05 | 1.91 | *Methanomicrobia* | *Methanosarcinales* (ANME2d) |
|  | 55.55 | 127,461 | 121,361 | 56 | 3.99 | 2.13 | *Methanomicrobia* | *Methanosarcinales* (*Methanosarcina*) |
|  | 58.70 | 159,324 | 145,886 | 78 | 8.53 | 2.88 | *Diaforarchaea* | DSPEG-I |
|  | 59.00 | 145,000 | 132,862 | 85 | 6.95 | 2.90 | *Methanomicrobia* | *Methanosarcinales* (*Methanosarcina*) |
|  | 67.00 | 285,525 | 262,655 | 78 | 4.59 | 2.50 | unknown *Bathyarchaeota* | MCG-8 |
| **Buor Khaya** | 4.72 | 94,240 | 84,493 | 69 | 5.67 | 2.42 | *Methanomicrobia* | *Methanosarcinales* (ANME2a-b) |
|  | 8.10 | 71,752 | 61,673 | 69 | 6.01 | 2.51 | unknown *Bathyarchaeota* | MCG-8 |
|  | 8.86 | 265,732 | 246,799 | 62 | 4.25 | 2.13 | unknown *Bathyarchaeota* | MCG-8 |
|  | 12.00 | 148,222 | 129,665 | 99 | 5.33 | 2.73 | unknown *Bathyarchaeota* | MCG-8 |
|  | 24.03-24.30 | 85,366 | 81,216 | 47 | 6.95 | 2.52 | unknown *Bathyarchaeota* | MCG-8 |
|  | 24.53-24.68 | 136,400 | 131,025 | 32 | 3.64 | 1.76 | *Methanomicrobia* | *Methanosarcinales* (*Methanosarcina*) |
|  | 30.31 | n.d. | n.d. | n.d. | n.d. | n.d. | n.d. | n.d. |
|  | 36.23 | n.d. | n.d. | n.d. | n.d. | n.d. | n.d. | n.d. |
|  | 37.41 | n.d. | n.d. | n.d. | n.d. | n.d. | n.d. | n.d. |
|  | 45.46 | 249,600 | 233,141 | 35 | 3.38 | 1.68 | *Diaforarchaea* | DSPEG-I |

***** Based on relative abundance of 16S rRNA sequences

n.d. not detected

**Table S4.** Oligonucleotides primer and probes used in this study.

| **Target gene** | **Primers** | **Sequence (5'-3')** | **Size [bp]** | **T (°C)** | **FA [%]** | **No. of**  **PCR Cycles** | **Reference** |  |
| --- | --- | --- | --- | --- | --- | --- | --- | --- |
| **Sequencing Illumina MiSeq** | | | | | | | | |
| *Bacteria*  16S rRNA | S-D-Bact-0341-a-S-17 | CCT ACG GGA GGC AGC AG | 464 | 55 |  | 30 | *28* | |
|  | S-D-Bact-0785-a-A-21 | GAC TAC HVG GGT ATC TAA TCC |  |  |  |  | *29* | |
| *Archaea*  16S rRNA | D-Arch-0020-a-S-19 | TTC CGG TTG ATC CYG CCR G | 956 | 55 |  | 40 | *30* | |
|  | S-D-Arch-0958-a-A-19 | YCC GGC GTT GAV TCC AAT T |  |  |  |  | *31* | |
| *Archaea*  16S rRNA | S-D-Arch-0349-a-S-17 | GYG CAS CAG KCG MGA AW | 457 | 56 |  | 35 | *32* | |
|  | S-D-Arch-0786-a-A-20 | GGA CTA CVS GGG TAT CTA AT |  |  |  |  |  |  |
| **Sequencing 454/ quantitative PCR** | | | | | | | | |
| Methanogens and ANME | mlas | GGT GGT GTM GGD TTC ACM CAR TA | 469 | 65-50 (touchdown)/ 55 (final); 57 (qPCR) |  | 15/ 15; 40 (qPCR) | *33* | |
|  | *mcrA*-rev | CGT TCA TBG CGT AGT TVG GRT AGT |  |  |  |  |  |  |
| **quantitative PCR** | | | | | | | | |
| sulfate-reducing *Bacteria* | DSRp2060F | CAA CAT CGT YCA YAC CCA GGG | 350 | 60 |  | 40 | *34* | |
|  | DSR4R | GTG TAG CAG TTA CCG CA |  |  |  |  | *35* | |
| ANME-2d | mcrA159F | AAA GTG CGG AGC AGC AAT CAC C | 186 | 60 |  | 40 | *36* | |
|  | mcrA345R | TCG TCC CAT TCC TGC TGC ATT GC |  |  |  |  |  |  |
| **16S rRNA clone libraries** | | | | | | | | |
| *Archaea*  16S rRNA | S-D-Arch-0008-a-S-16 | TCC GGT TGA TCC TGC C | 638 | 59 |  | 30 | *37* | |
| ANME-2a  16S rRNA | ANME-2a-647 | TCT TCC GGT CCC AAG CCT |  |  |  |  | *38* | |
| **16S rRNA probes** | | | | | | | | |
| ANME-2a | ANME-2a-647 | TCT TCC GGT CCC AAG CCT |  |  | 50 |  | *38* | |
| *Desulfosarcina-Desulfococcus* | DSS658 | TCC ACT TCC CTC TCC CAT |  |  | 50 |  | *39* | |

**Table S5.** Barcode sequences for Illumina MiSeq sequencing used in this study.

| **Barcode_ID** | **Barcode sequence** |
| --- | --- |
| 00 | ACACGT |
| 01 | ACGTAC |
| 02 | ACTGCA |
| 03 | AGAGTC |
| 04 | AGCTGA |
| 05 | AGTCAG |
| 06 | ATATCG |
| 07 | ATCGAT |
| 08 | ATGCTA |
| 09 | CACAGT |
| 10 | CGATAT |
| 11 | CATGAC |
| 12 | GACTAG |
| 13 | GAGATC |
| 14 | GATCGA |
| 15 | TATACG |
| 16 | TCTCTC |
| 17 | TGCATG |
| 18 | TGACGT |
| 19 | TGTGAC |

**Table S6. Barcode sequences of 454 sequencing used in this study.**

| **MID_ID** | **MID sequence** |
| --- | --- |
| MID-01 | ACGAGTGCGT |
| MID-02 | ACGCTCGACA |
| MID-03 | AGACGCACTC |
| MID-04 | AGCACTGTAG |
| MID-05 | ATCAGACACG |
| MID-06 | ATATCGCGAG |
| MID-07 | CGTGTCTCTA |
| MID-08 | CTCGCGTGTC |
| MID-11 | TGATACGTCT |
| MID-13 | CATAGTAGTG |
| MID-14 | CGAGAGATAC |
| MID-15 | ATACGACGTA |
| MID-16 | TCACGTACTA |
| MID-17 | CGTCTAGTAC |
| MID-18 | TCTACGTAGC |
| MID-20 | ACGACTACAG |

**Table S7.** Percentage proportion of phospholipid fatty acids (PLFAs) in different depths of the submarine permafrost core BK2.

|  | **depth [m bsf]** | | | | | |
| --- | --- | --- | --- | --- | --- | --- |
| **PLFAs [%]** | **8.10** | **12.85** | **24.03** | **24.30** | **24.60** | **24.70** |
| **12:0** | 1.29 | 1.36 | 2.71 | 1.93 | 1.49 | 1.07 |
| ***iso*14:0** | 1.20 | 0 | 0 | 0 | 0 | 0 |
| **14:0** | 4.20 | 3.06 | 4.61 | 2.94 | 4.19 | 1.86 |
| ***iso*15:0** | 4.83 | 1.75 | 0.89 | 0.55 | 1.45 | 1.86 |
| ***ai*15:0** | 7.27 | 1.92 | 1.31 | 0.95 | 1.32 | 1.15 |
| **15:0** | 1.90 | 1.87 | 2.12 | 1.26 | 1.92 | 1.12 |
| ***iso*16:0** | 1.15 | 0.77 | 0 | 0 | 0 | 0 |
| **16:1ω7c** | 7.94 | 1.73 | 3.53 | 3.15 | 2.64 | 4.13 |
| **16:1ω5** | 1.73 | 0 | 0 | 0 | 0 | 0 |
| **16:0** | 26.30 | 27.86 | 32.26 | 31.21 | 32.00 | 30.72 |
| **10Me-16:0** | 1.08 | 0.00 | 0.00 | 0 | 0 | 0 |
| ***iso*17:0** | 0.63 | 0 | 0 | 0 | 0 | 0 |
| ***ai*17:0** | 0.99 | 1.52 | 3.67 | 0 | 0 | 1.35 |
| ***cy*17:0ω7,8** | 1.73 | 0.00 | 0 | 0 | 0 | 0 |
| **17:0** | 1.78 | 2.85 | 2.02 | 2.03 | 2.04 | 1.32 |
| **18:2ω9,12** | 2.02 | 2.54 | 0 | 0 | 0 | 0 |
| **18:1ω9** | 4.77 | 6.39 | 10.73 | 8.62 | 9.44 | 3.23 |
| **18:1ω7c** | 10.54 | 3.25 | 5.38 | 8.62 | 4.94 | 4.54 |
| **18:0** | 18.66 | 43.13 | 30.78 | 38.73 | 38.58 | 47.63 |

Blue shaded area represents SMTZ sediment layers.

**References Supplement**

1. Martin, M. Cutadapt removes adapter sequences from high-throughput sequencing reads. *EMBnet.journal* **17,** 10–12 (2011).

2. Zhang, J., Kobert, K., Flouri, T. & Stamatakis, A. PEAR: a fast and accurate Illumina Paired-End reAd mergeR. *Bioinformatics* **30,** 614–620 (2014).

3. Bolger, A. M., Lohse, M. & Usadel, B. Trimmomatic: a flexible trimmer for Illumina sequence data. *Bioinformatics* **30,** 2114–2120 (2014).

4. Haas, B. J. *et al.* Chimeric 16S rRNA sequence formation and detection in Sanger and 454-pyrosequenced PCR amplicons. *Genome Res.* **21,** 494–504 (2011).

5. Edgar, R. C. Search and clustering orders of magnitude faster than BLAST. *Bioinformatics* **26,** 2460–2461 (2010).

6. McDonald, D. *et al.* An improved Greengenes taxonomy with explicit ranks for ecological and evolutionary analyses of bacteria and archaea. *ISME J* **6,** 610–618 (2012).

7. Caporaso, J. G. *et al.* QIIME allows analysis of high-throughput community sequencing data. *Nature Methods* **7,** 335–336 (2010).

8. Rinke, C. *et al.* Insights into the phylogeny and coding potential of microbial dark matter. *Nature* **499,** 431–437 (2013).

9. Castelle, C. J. *et al.* Genomic expansion of domain archaea highlights roles for organisms from new phyla in anaerobic carbon cycling. *Current Biology* **25,** 690–701 (2015).

10. Adam, P. S., Borrel, G., Brochier-Armanet, C. & Gribaldo, S. The growing tree of Archaea: new perspectives on their diversity, evolution and ecology. *ISME J.* (2017). doi:10.1038/ismej.2017.122

11. Schloss, P. D. *et al.* Introducing mothur: Open-source, platform-independent, community-supported software for describing and comparing microbial communities. *Appl. Environ. Microbiol.* **75,** 7537–7541 (2009).

12. Yang, S., Liebner, S., Alawi, M., Ebenhöh, O. & Wagner, D. Taxonomic database and cut-off value for processing *mcrA* gene 454 pyrosequencing data by MOTHUR. *Journal of Microbiological Methods* **103,** 3–5 (2014).

13. Pruitt, K. D., Tatusova, T. & Maglott, D. R. NCBI Reference Sequence (RefSeq): a curated non-redundant sequence database of genomes, transcripts and proteins. *Nucleic Acids Res* **33,** D501–D504 (2005).

14. Ludwig, W. *et al.* ARB: a software environment for sequence data. *Nucl Acids Res* **32,** 1363–1371 (2004).

15. Hunger, S. *et al.* Competing formate- and carbon dioxide-utilizing prokaryotes in an anoxic methane-emitting fen soil. *Appl. Environ. Microbiol.* **77,** 3773–3785 (2011).

16. Ishii, K., Mußmann, M., MacGregor, B. J. & Amann, R. An improved fluorescence *in situ* hybridization protocol for the identification of bacteria and archaea in marine sediments. *FEMS Microbiology Ecology* **50,** 203–213 (2004).

17. Teira, E., Reinthaler, T., Pernthaler, A., Pernthaler, J. & Herndl, G. J. Combining catalyzed reporter deposition-fluorescence *in situ* hybridization and microautoradiography to detect substrate utilization by *Bacteria* and *Archaea* in the deep ocean. *Appl Environ Microbiol.* **70,** 4411–4414 (2004).

18. Zink, K.-G. & Mangelsdorf, K. Efficient and rapid method for extraction of intact phospholipids from sediments combined with molecular structure elucidation using LC–ESI-MS–MS analysis. *Anal. Bioanal. Chem.* **380,** 798–812 (2004).

19. Müller, K.-D., Husmann, H. & Nalik, H. P. A new and rapid method for the assay of bacterial fatty acids using high resolution capillary gas chromatography and trimethylsulfonium hydroxide. *Zentralblatt Für Bakteriol.* **274,** 174–182 (1990).

20. Schouten, S., Huguet, C., Hopmans, E. C., Kienhuis, M. V. M. & Sinninghe Damsté, J. S. Analytical methodology for TEX86 paleothermometry by high-performance liquid chromatography/atmospheric pressure chemical ionization-mass spectrometry. *Anal. Chem.* **79,** 2940–2944 (2007).

21. Bischoff, J., Mangelsdorf, K., Schwamborn, G. & Wagner, D. Impact of lake-level and climate changes on microbial communities in a terrestrial permafrost sequence of the El’gygytgyn Crater, Far East Russian Arctic. *Permafr. Periglac. Process.* **25,** 107–116 (2014).

22. Kallmeyer J., Pockalny R., Adhikari R.R., Smith D.C., D’Hondt S. Global distribution of microbial abundance and biomass in subseafloor sediment. *Proc Natl Acad Sci*. **109,** 16213-16216 (2012).

23. Segarra, K. E. A. *et al.* High rates of anaerobic methane oxidation in freshwater wetlands reduce potential atmospheric methane emissions. *Nat. Commun.* **6,** (2015).

24. Bannert, A. *et al.* Anaerobic oxidation of methane in grassland soils used for cattle husbandry. *Biogeosciences* **9,** 3891–3899 (2012).

25. Elvert, M., Boetius, A., Knittel, K. & Jørgensen, B. B. Characterization of specific membrane fatty acids as chemotaxonomic markers for sulfate-reducing bacteria involved in anaerobic oxidation of methane. *Geomicrobiol. J.* **20,** 403–419 (2003).

26. Stapel, J. G. *et al.* Microbial lipid signatures and substrate potential of organic matter in permafrost deposits: Implications for future greenhouse gas production. *J. Geophys. Res. Biogeosciences* **121,** 2016JG003483 (2016).

27. Weijers, J. W. H., Lim, K. L. H., Aquilina, A., Sinninghe Damsté, J. S. & Pancost, R. D. Biogeochemical controls on glycerol dialkyl glycerol tetraether lipid distributions in sediments characterized by diffusive methane flux. *Geochem. Geophys. Geosyst.* **12,** Q10010 (2011).

28. Muyzer, G., Waal, E. C. de & Uitterlinden, A. G. Profiling of complex microbial populations by denaturing gradient gel electrophoresis analysis of polymerase chain reaction-amplified genes coding for 16S rRNA. *Appl Environ Microbiol.* **59,** 695–700 (1993).

29. Herlemann, D. P. *et al.* Transitions in bacterial communities along the 2000 km salinity gradient of the Baltic Sea. *The ISME journal* **5,** 1571–9 (2011).

30. Massana, R., Murray, A. E., Preston, C. M. & DeLong, E. F. Vertical distribution and phylogenetic characterization of marine planktonic *Archaea* in the Santa Barbara Channel. *Appl Environ Microbiol.* **63,** 50–56 (1997).

31. DeLong, E. F. Archaea in coastal marine environments. *PNAS* **89,** 5685–5689 (1992).

32. Takai, K., Horikoshi, K. & Takai, K. E. N. Rapid detection and quantification of members of the archaeal community by quantitative PCR using fluorogenic probes. *Applied and Environmental Microbiology* **66,** 5066–5072 (2000).

33. Steinberg, L. M. & Regan, J. M. Phylogenetic comparison of the methanogenic communities from an acidic, oligotrophic fen and an anaerobic digester treating municipal wastewater sludge. *Applied and Environmental Microbiology* **74,** 6663–6671 (2008).

34. Geets, J. *et al.* DsrB gene-based DGGE for community and diversity surveys of sulfate-reducing bacteria. *Journal of Microbiological Methods* **66,** 194–205 (2006).

35. Wagner, M., Roger, A. J., Flax, J. L., Brusseau, G. A. & Stahl, D. A. Phylogeny of dissimilatory sulfite reductases supports an early origin of sulfate respiration. *J. Bacteriol.* **180,** 2975–2982 (1998).

36. Vaksmaa, A., Jetten, M. S. M., Ettwig, K. F. & Lüke, C. *McrA* primers for the detection and quantification of the anaerobic archaeal methanotroph ‘*Candidatus* Methanoperedens nitroreducens’. *Appl. Microbiol. Biotechnol.* **101,** 1631–1641 (2017).

37. Teske, A. *et al.* Microbial diversity of hydrothermal sediments in the Guaymas Basin: Evidence for anaerobic methanotrophic communities. *Appl. Environ. Microbiol.* **68,** 1994–2007 (2002).

38. Knittel, K., Lösekann, T., Boetius, A., Kort, R. & Amann, R. Diversity and distribution of methanotrophic archaea at Cold Seeps. *Appl. Environ. Microbiol.* **71,** 467–479 (2005).

39. Manz, W., Eisenbrecher, M., Neu, T. R. & Szewzyk, U. Abundance and spatial organization of Gram-negative sulfate-reducing bacteria in activated sludge investigated by *in situ* probing with specific 16S rRNA targeted oligonucleotides. *FEMS Microbiology Ecology* **25,** 43–61 (1998).
