# Supplementary material for: Anaerobic methanotrophic communities thrive in deep submarine permafrost
Source: Sci Rep. 2018 Jan 22;8:1291. doi: 10.1038/s41598-018-19505-9 (PMC5778128; doi:10.1038/s41598-018-19505-9)
Supplement: Supplementary file 2 — Supplementary Figure S1 [file 41598_2018_19505_MOESM2_ESM.pdf]

Title

Anaerobic methanotrophic communities thrive in deep submarine permafrost

Authors

Matthias Winkel[1,*], Julia Mitzscherling[1], Pier P. Overduin[2], Fabian Horn[1], Maria Winterfeld[3], Ruud Rijkers[1], Mikhail N. Grigoriev[4], Christian Knoblauch[5], Kai Mangelsdorf[6], Dirk Wagner[1], and Susanne Liebner[1]

Affiliations

[1]GFZ German Research Centre for Geosciences, Helmholtz Centre Potsdam, Section 5.3 Geomicrobiology, 14473 Potsdam, Germany

[2]Alfred Wegener Institute, Helmholtz Centre for Polar and Marine Research, Periglacial Research, 14473 Potsdam, Germany

[3]Alfred Wegener Institute, Helmholtz Centre for Polar and Marine Research, Marine Geochemistry, 27570 Bremerhaven, Germany

[4]Mel’nikov Permafrost Institute, SB RAS, Yakutsk, 677010 Russia

[5]Institute of Soil Science, Universität Hamburg, 20146 Hamburg, Germany

[6]GFZ German Research Centre for Geosciences, Helmholtz Centre Potsdam, Section 3.2 Organic Geochemistry, 14473 Potsdam, Germany

*Corresponding author

E-mail: mwinkel@gfz-potsdam.de

Address: Telegrafenberg, building F, room 357, 14473 Potsdam, Germany

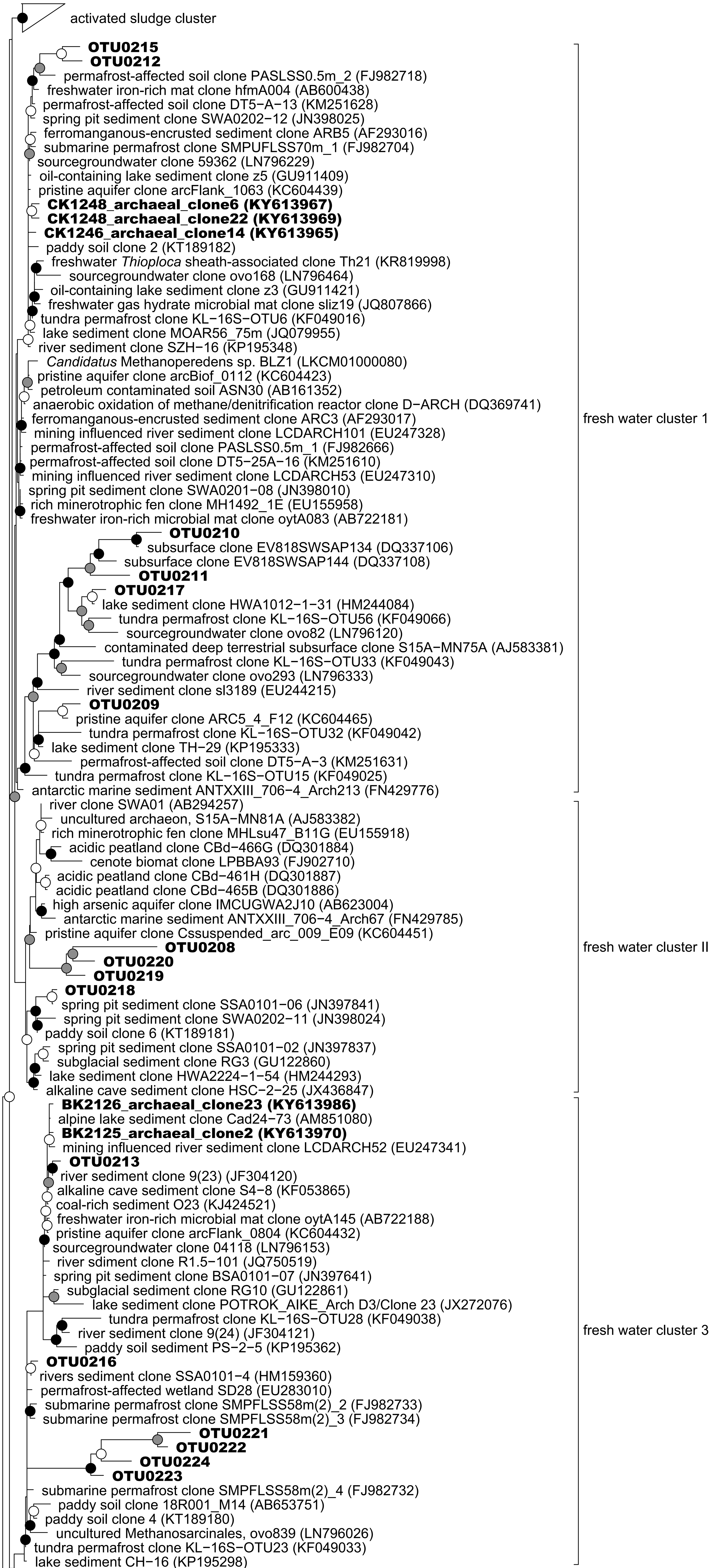
activated sludge cluster
OTU0215
OTU0212
permafrost-affected soil clone PASLSS0.5m_2 (FJ982718)
freshwater iron-rich mat clone hfmA004 (AB600438)
permafrost-affected soil clone DT5−A−13 (KM251628)
spring pit sediment clone SWA0202−12 (JN398025)
ferromanganous-encrusted sediment clone ARB5 (AF293016)
submarine permafrost clone SMPUFLSS70m_1 (FJ982704)
sourcegroundwater clone 59362 (LN796229)
oil-containing lake sediment clone z5 (GU911409)
pristine aquifer clone arcFlank_1063 (KC604439)
CK1248_archaeal_clone6 (KY613967)
CK1248_archaeal_clone22 (KY613969)
CK1246_archaeal_clone14 (KY613965)
paddy soil clone 2 (KT189182)
freshwater Thioploca sheath-associated clone Th21 (KR819998)
sourcegroundwater clone ovo168 (LN796464)
oil-containing lake sediment clone z3 (GU911421)
freshwater gas hydrate microbial mat clone sliz19 (JQ807866)
tundra permafrost clone KL−16S−OTU6 (KF049016)
lake sediment clone MOAR56_75m (JQ079955)
river sediment clone SZH−16 (KP195348)
Candidatus Methanoperedens sp. BLZ1 (LKCM01000080)
pristine aquifer clone arcBiof_0112 (KC604423)
petroleum contaminated soil ASN30 (AB161352)
anaerobic oxidation of methane/denitrification reactor clone D−ARCH (DQ369741)
ferromanganous-encrusted sediment clone ARC3 (AF293017)
mining influenced river sediment clone LCDARCH101 (EU247328)
permafrost-affected soil clone PASLSS0.5m_1 (FJ982666)
permafrost-affected soil clone DT5−25A−16 (KM251610)
mining influenced river sediment clone LCDARCH53 (EU247310)
spring pit sediment clone SWA0201−08 (JN398010)
rich minerotrophic fen clone MH1492_1E (EU155958)
freshwater iron-rich microbial mat clone oytA083 (AB722181)
OTU0210
subsurface clone EV818SWSAP134 (DQ337106)
subsurface clone EV818SWSAP144 (DQ337108)
OTU0211
OTU0217
lake sediment clone HWA1012−1−31 (HM244084)
tundra permafrost clone KL−16S−OTU56 (KF049066)
sourcegroundwater clone ovo82 (LN796120)
contaminated deep terrestrial subsurface clone S15A−MN75A (AJ583381)
tundra permafrost clone KL−16S−OTU33 (KF049043)
sourcegroundwater clone ovo293 (LN796333)
river sediment clone sl3189 (EU244215)
OTU0209
pristine aquifer clone ARC5_4_F12 (KC604465)
tundra permafrost clone KL−16S−OTU32 (KF049042)
lake sediment clone TH−29 (KP195333)
permafrost-affected soil clone DT5−A−3 (KM251631)
tundra permafrost clone KL−16S−OTU15 (KF049025)
antarctic marine sediment ANTXXIII_706−4_Arch213 (FN429776)
fresh water cluster 1
river clone SWA01 (AB294257)
uncultured archaeon, S15A−MN81A (AJ583382)
rich minerotrophic fen clone MHLsu47_B11G (EU155918)
acidic peatland clone CBd−466G (DQ301884)
cenote biomat clone LPBBA93 (FJ902710)
acidic peatland clone CBd−461H (DQ301887)
acidic peatland clone CBd−465B (DQ301886)
high arsenic aquifer clone IMCUGWA2J10 (AB623004)
antarctic marine sediment ANTXXIII_706−4_Arch67 (FN429785)
pristine aquifer clone Cssuspended_arc_009_E09 (KC604451)
OTU0208
OTU0220
OTU0219
OTU0218
spring pit sediment clone SSA0101−06 (JN397841)
spring pit sediment clone SWA0202−11 (JN398024)
paddy soil clone 6 (KT189181)
spring pit sediment clone SSA0101−02 (JN397837)
subglacial sediment clone RG3 (GU122860)
lake sediment clone HWA2224−1−54 (HM244293)
alkaline cave sediment clone HSC−2−25 (JX436847)
fresh water cluster II
BK2126_archaeal_clone23 (KY613986)
alpine lake sediment clone Cad24−73 (AM851080)
BK2125_archaeal_clone2 (KY613970)
mining influenced river sediment clone LCDARCH52 (EU247341)
OTU0213
river sediment clone 9(23) (JF304120)
alkaline cave sediment clone S4−8 (KF053865)
coal-rich sediment O23 (KJ424521)
freshwater iron-rich microbial mat clone oytA145 (AB722188)
pristine aquifer clone arcFlank_0804 (KC604432)
sourcegroundwater clone 04118 (LN796153)
river sdiment clone R1.5−101 (JQ750519)
spring pit sediment clone BSA0101−07 (JN397641)
subglacial sediment clone RG10 (GU122861)
lake sediment clone POTROK_AIKE_Arch D3/Clone 23 (JX272076)
tundra permafrost clone KL−16S−OTU28 (KF049038)
river sediment clone 9(24) (JF304121)
paddy soil sediment PS−2−5 (KP195362)
OTU0216
rivers sediment clone SSA0101−4 (HM159360)
permafrost-affected wetland SD28 (EU283010)
submarine permafrost clone SMPFLSS58m(2)_2 (FJ982733)
submarine permafrost clone SMPFLSS58m(2)_3 (FJ982734)
OTU0221
OTU0222
OTU0224
OTU0223
submarine permafrost clone SMPFLSS58m(2)_4 (FJ982732)
paddy soil clone 18R001_M14 (AB653751)
paddy soil clone 4 (KT189180)
uncultured Methanosarcinales, ovo839 (LN796026)
tundra permafrost clone KL−16S−OTU23 (KF049033)
lake sediment CH−16 (KP195298)
fresh water cluster 3

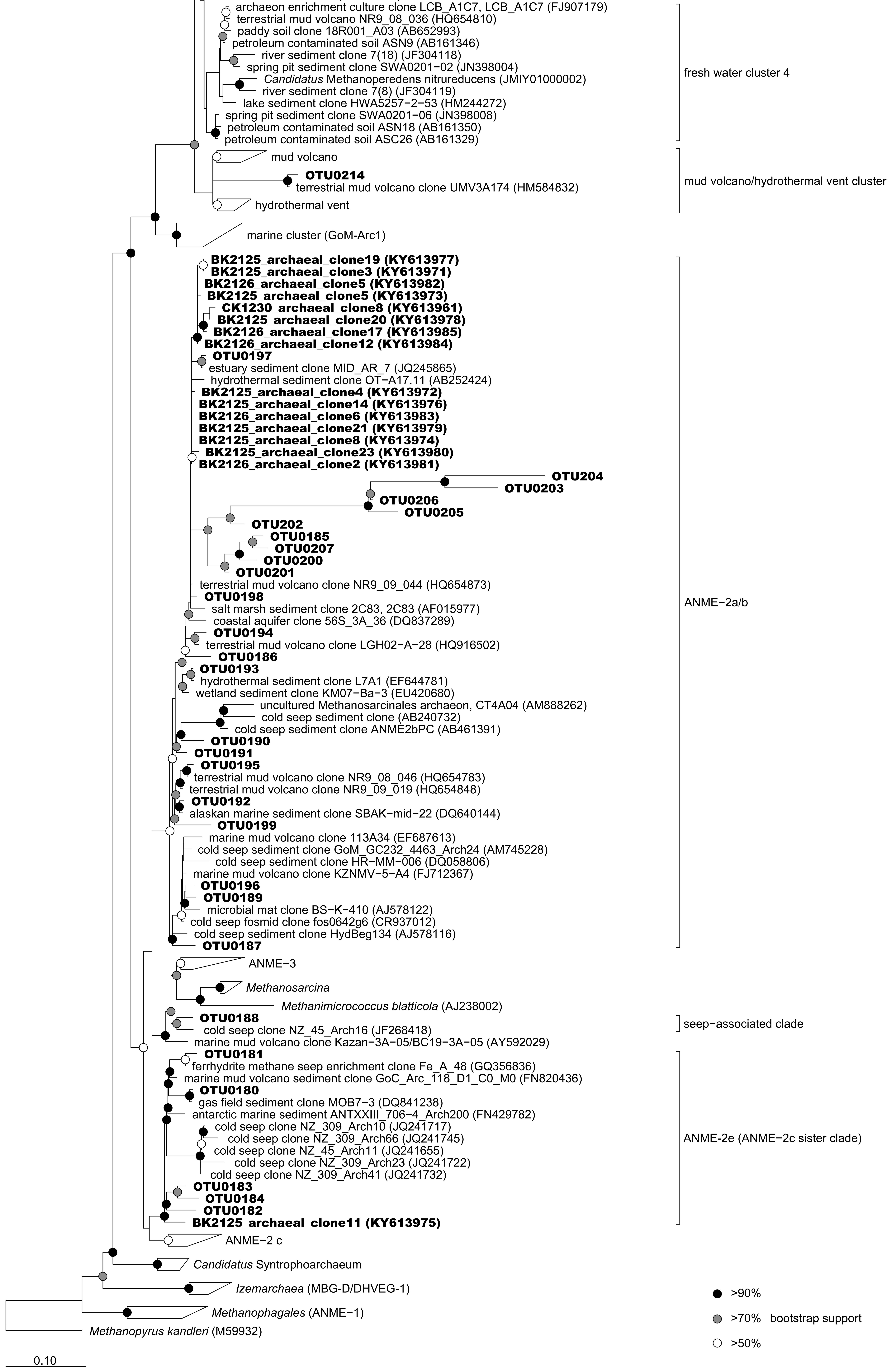
archaeon enrichment culture clone LCB_A1C7, LCB_A1C7 (FJ907179)
terrestrial mud volcano NR9_08_036 (HQ654810)
paddy soil clone 18R001_A03 (AB652993)
petroleum contaminated soil ASN9 (AB161346)
river sediment clone 7(18) (JF304118)
spring pit sediment clone SWA0201-02 (JN398004)
Candidatus Methanoperedens nitrureducens (JMIY01000002)
river sediment clone 7(8) (JF304119)
lake sediment clone HWA5257-2-53 (HM244272)
spring pit sediment clone SWA0201-06 (JN398008)
petroleum contaminated soil ASN18 (AB161350)
petroleum contaminated soil ASC26 (AB161329)
fresh water cluster 4
mud volcano
OTU0214
terrestrial mud volcano clone UMV3A174 (HM584832)
hydrothermal vent
mud volcano/hydrothermal vent cluster
marine cluster (GoM-Arc1)
BK2125_archaeal_clone19 (KY613977)
BK2125_archaeal_clone3 (KY613971)
BK2126_archaeal_clone5 (KY613982)
BK2125_archaeal_clone5 (KY613973)
CK1230_archaeal_clone8 (KY613961)
BK2125_archaeal_clone20 (KY613978)
BK2126_archaeal_clone17 (KY613985)
BK2126_archaeal_clone12 (KY613984)
OTU0197
estuary sediment clone MID_AR_7 (JQ245865)
hydrothermal sediment clone OT-A17.11 (AB252424)
BK2125_archaeal_clone4 (KY613972)
BK2125_archaeal_clone14 (KY613976)
BK2126_archaeal_clone6 (KY613983)
BK2125_archaeal_clone21 (KY613979)
BK2125_archaeal_clone8 (KY613974)
BK2125_archaeal_clone23 (KY613980)
BK2126_archaeal_clone2 (KY613981)
OTU204
OTU0203
OTU0206
OTU0205
OTU202
OTU0185
OTU0207
OTU0200
OTU0201
terrestrial mud volcano clone NR9_09_044 (HQ654873)
OTU0198
salt marsh sediment clone 2C83, 2C83 (AF015977)
coastal aquifer clone 56S_3A_36 (DQ837289)
OTU0194
terrestrial mud volcano clone LGH02-A-28 (HQ916502)
OTU0186
OTU0193
hydrothermal sediment clone L7A1 (EF644781)
wetland sediment clone KM07-Ba-3 (EU420680)
uncultured Methanosarcinales archaeon, CT4A04 (AM888262)
cold seep sediment clone (AB240732)
cold seep sediment clone ANME2bPC (AB461391)
OTU0190
OTU0191
OTU0195
terrestrial mud volcano clone NR9_08_046 (HQ654783)
terrestrial mud volcano clone NR9_09_019 (HQ654848)
OTU0192
alaskan marine sediment clone SBAK-mid-22 (DQ640144)
OTU0199
marine mud volcano clone 113A34 (EF687613)
cold seep sediment clone GoM_GC232_4463_Arch24 (AM745228)
cold seep sediment clone HR-MM-006 (DQ058806)
marine mud volcano clone KZNMV-5-A4 (FJ712367)
OTU0196
OTU0189
microbial mat clone BS-K-410 (AJ578122)
cold seep fosmid clone fos0642g6 (CR937012)
cold seep sediment clone HydBeg134 (AJ578116)
OTU0187
ANME-2a/b
ANME-3
Methanosarcina
Methanimicrococcus blatticola (AJ238002)
OTU0188
cold seep clone NZ_45_Arch16 (JF268418)
marine mud volcano clone Kazan-3A-05/BC19-3A-05 (AY592029)
seep-associated clade
OTU0181
ferrhydrite methane seep enrichment clone Fe_A_48 (GQ356836)
marine mud volcano sediment clone GoC_Arc_118_D1_C0_M0 (FN820436)
OTU0180
gas field sediment clone MOB7-3 (DQ841238)
antarctic marine sediment ANTXXIII_706-4_Arch200 (FN429782)
cold seep clone NZ_309_Arch10 (JQ241717)
cold seep clone NZ_309_Arch66 (JQ241745)
cold seep clone NZ_45_Arch11 (JQ241655)
cold seep clone NZ_309_Arch23 (JQ241722)
cold seep clone NZ_309_Arch41 (JQ241732)
OTU0183
OTU0184
OTU0182
BK2125_archaeal_clone11 (KY613975)
ANME-2e (ANME-2c sister clade)
ANME-2 c
Candidatus Syntrophoarchaeum
Izemarchaea (MBG-D/DHVEG-1)
Methanophagales (ANME-1)
Methanopyrus kandleri (M59932)
0.10
>90%
>70% bootstrap support
>50%
