# Supplementary material for: Anaerobic methanotrophic communities thrive in deep submarine permafrost
Source: Sci Rep. 2018 Jan 22;8:1291. doi: 10.1038/s41598-018-19505-9 (PMC5778128; doi:10.1038/s41598-018-19505-9)
Supplement: Supplementary file 3 — Supplementary Figure S2 [file 41598_2018_19505_MOESM3_ESM.pdf]

Title

Anaerobic methanotrophic communities thrive in deep submarine permafrost

Authors

Matthias Winkel[1,*], Julia Mitzscherling[1], Pier P. Overduin[2], Fabian Horn[1], Maria Winterfeld[3], Ruud Rijkers[1], Mikhail N. Grigoriev[4], Christian Knoblauch[5], Kai Mangelsdorf[6], Dirk Wagner[1], and Susanne Liebner[1]

Affiliations

[1]GFZ German Research Centre for Geosciences, Helmholtz Centre Potsdam, Section 5.3 Geomicrobiology, 14473 Potsdam, Germany

[2]Alfred Wegener Institute, Helmholtz Centre for Polar and Marine Research, Periglacial Research, 14473 Potsdam, Germany

[3]Alfred Wegener Institute, Helmholtz Centre for Polar and Marine Research, Marine Geochemistry, 27570 Bremerhaven, Germany

[4]Mel'nikov Permafrost Institute, SB RAS, Yakutsk, 677010 Russia

[5]Institute of Soil Science, Universität Hamburg, 20146 Hamburg, Germany

[6]GFZ German Research Centre for Geosciences, Helmholtz Centre Potsdam, Section 3.2 Organic Geochemistry, 14473 Potsdam, Germany

*Corresponding author

E-mail: mwinkel@gfz-potsdam.de

Address: Telegrafenberg, building F, room 357, 14473 Potsdam, Germany

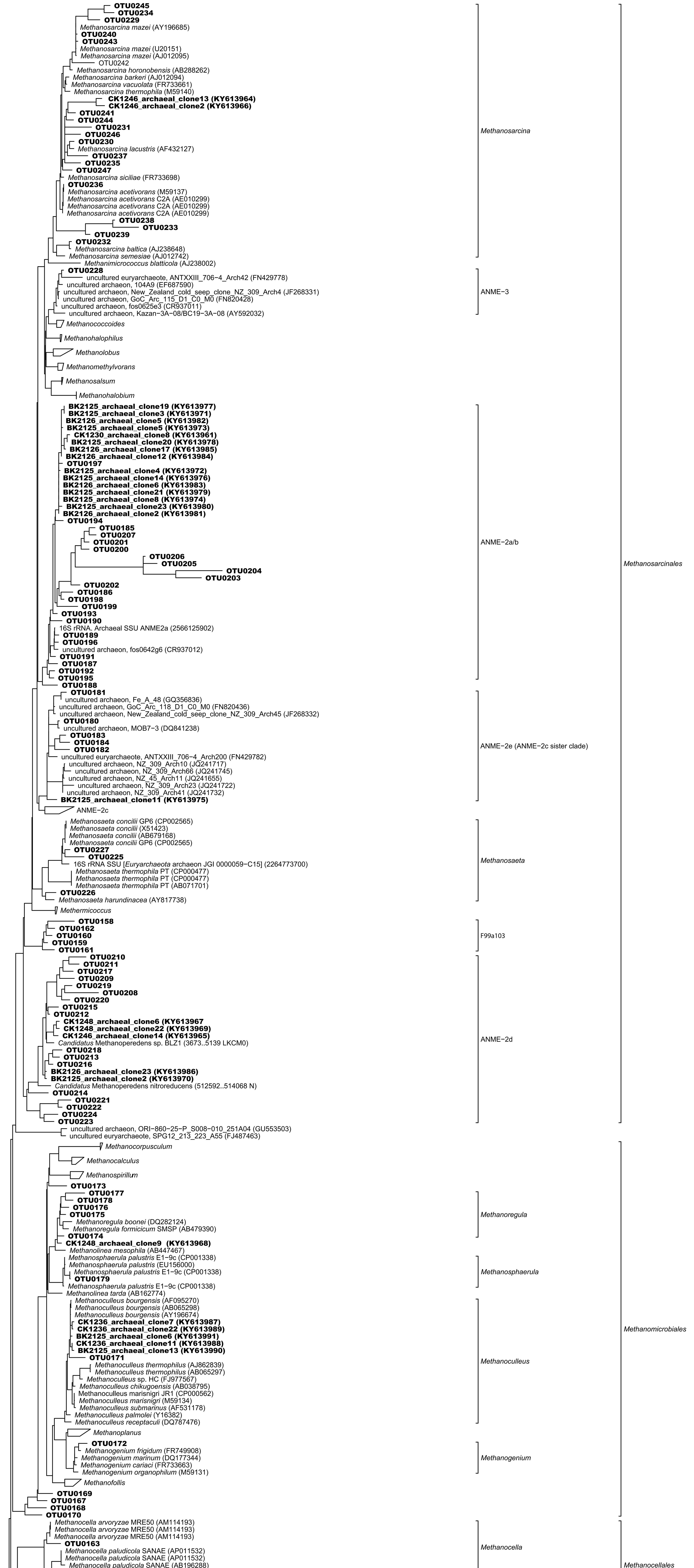

OTU0245
OTU0234
OTU0229
Methanosarcina mazei (AY196685)
OTU0240
OTU0243
Methanosarcina mazei (U20151)
Methanosarcina mazei (AJ012095)
OTU0242
Methanosarcina horonobensis (AB288262)
Methanosarcina barkeri (AJ012094)
Methanosarcina vacuolata (FR733661)
Methanosarcina thermophila (M59140)
CK1246_archaeal_clone13 (KY613964)
CK1246_archaeal_clone2 (KY613966)
OTU0241
OTU0244
OTU0231
OTU0246
OTU0230
Methanosarcina lacustris (AF432127)
OTU0237
OTU0235
OTU0247
Methanosarcina siciliae (FR733698)
OTU0236
Methanosarcina acetivorans (M59137)
Methanosarcina acetivorans C2A (AE010299)
Methanosarcina acetivorans C2A (AE010299)
Methanosarcina acetivorans C2A (AE010299)
OTU0238
OTU0233
OTU0239
OTU0232
Methanosarcina baltica (AJ238648)
Methanosarcina semesiae (AJ012742)
Methanimicrococcus blatticola (AJ238002)
OTU0228
uncultured euryarchaeote, ANTXXIII_706−4_Arch42 (FN429778)
uncultured archaeon, 104A9 (EF687590)
uncultured archaeon, New_Zealand_cold_seep_clone_NZ_309_Arch4 (JF268331)
uncultured archaeon, GoC_Arc_115_D1_C0_M0 (FN820428)
uncultured archaeon, fos0625e3 (CR937011)
uncultured archaeon, Kazan−3A−08/BC19−3A−08 (AY592032)
Methanococcoides
Methanohalophilus
Methanolobus
Methanomethylvorans
Methanosalsum
Methanohalobium
BK2125_archaeal_clone19 (KY613977)
BK2125_archaeal_clone3 (KY613971)
BK2126_archaeal_clone5 (KY613982)
BK2125_archaeal_clone5 (KY613973)
CK1230_archaeal_clone8 (KY613961)
BK2125_archaeal_clone20 (KY613978)
BK2126_archaeal_clone17 (KY613985)
BK2126_archaeal_clone12 (KY613984)
OTU0197
BK2125_archaeal_clone4 (KY613972)
BK2125_archaeal_clone14 (KY613976)
BK2126_archaeal_clone6 (KY613983)
BK2125_archaeal_clone21 (KY613979)
BK2125_archaeal_clone8 (KY613974)
BK2125_archaeal_clone23 (KY613980)
BK2126_archaeal_clone2 (KY613981)
OTU0194
OTU0185
OTU0207
OTU0201
OTU0200
OTU0206
OTU0205
OTU0204
OTU0203
OTU0202
OTU0186
OTU0198
OTU0199
OTU0193
OTU0190
16S rRNA, Archaeal SSU ANME2a (2566125902)
OTU0189
OTU0196
uncultured archaeon, fos0642g6 (CR937012)
OTU0191
OTU0187
OTU0192
OTU0195
OTU0188
OTU0181
uncultured archaeon, Fe_A_48 (GQ356836)
uncultured archaeon, GoC_Arc_118_D1_C0_M0 (FN820436)
uncultured archaeon, New_Zealand_cold_seep_clone_NZ_309_Arch45 (JF268332)
OTU0180
uncultured archaeon, MOB7−3 (DQ841238)
OTU0183
OTU0184
OTU0182
uncultured euryarchaeote, ANTXXIII_706−4_Arch200 (FN429782)
uncultured archaeon, NZ_309_Arch10 (JQ241717)
uncultured archaeon, NZ_309_Arch66 (JQ241745)
uncultured archaeon, NZ_45_Arch11 (JQ241655)
uncultured archaeon, NZ_309_Arch23 (JQ241722)
uncultured archaeon, NZ_309_Arch41 (JQ241732)
BK2125_archaeal_clone11 (KY613975)
ANME−2c
Methanosaeta concilii GP6 (CP002565)
Methanosaeta concilii (X51423)
Methanosaeta concilii (AB679168)
Methanosaeta concilii GP6 (CP002565)
OTU0227
OTU0225
16S rRNA SSU [Euryarchaeota archaeon JGI 0000059−C15] (2264773700)
Methanosaeta thermophila PT (CP000477)
Methanosaeta thermophila PT (CP000477)
Methanosaeta thermophila PT (AB071701)
OTU0226
Methanosaeta harundinacea (AY817738)
Methermicoccus
OTU0158
OTU0162
OTU0160
OTU0159
OTU0161
OTU0210
OTU0211
OTU0217
OTU0209
OTU0219
OTU0208
OTU0220
OTU0215
OTU0212
CK1248_archaeal_clone6 (KY613967)
CK1248_archaeal_clone22 (KY613969)
CK1246_archaeal_clone14 (KY613965)
Candidatus Methanoperedens sp. BLZ1 (3673..5139 LKCM0)
OTU0218
OTU0213
OTU0216
BK2126_archaeal_clone23 (KY613986)
BK2125_archaeal_clone2 (KY613970)
Candidatus Methanoperedens nitroreducens (512592..514068 N)
OTU0214
OTU0221
OTU0222
OTU0224
OTU0223
uncultured archaeon, ORI−860−25−P_S008−010_251A04 (GU553503)
uncultured euryarchaeote, SPG12_213_223_A55 (FJ487463)
Methanocorpusculum
Methanocalculus
Methanospirillum
OTU0173
OTU0177
OTU0178
OTU0176
OTU0175
Methanoregula boonei (DQ282124)
Methanoregula formicicum SMSP (AB479390)
OTU0174
CK1248_archaeal_clone9 (KY613968)
Methanolinea mesophila (AB447467)
Methanosphaerula palustris E1−9c (CP001338)
Methanosphaerula palustris (EU156000)
Methanosphaerula palustris E1−9c (CP001338)
OTU0179
Methanosphaerula palustris E1−9c (CP001338)
Methanolinea tarda (AB162774)
Methanoculleus bourgensis (AF095270)
Methanoculleus bourgensis (AB065298)
Methanoculleus bourgensis (AY196674)
CK1236_archaeal_clone7 (KY613987)
CK1236_archaeal_clone22 (KY613989)
BK2125_archaeal_clone6 (KY613991)
CK1236_archaeal_clone11 (KY613988)
BK2125_archaeal_clone13 (KY613990)
OTU0171
Methanoculleus thermophilus (AJ862839)
Methanoculleus thermophilus (AB065297)
Methanoculleus sp. HC (FJ977567)
Methanoculleus chikugoensis (AB038795)
Methanoculleus marisnigri JR1 (CP000562)
Methanoculleus marisnigri (M59134)
Methanoculleus submarinus (AF531178)
Methanoculleus palmolei (Y16382)
Methanoculleus receptaculi (DQ787476)
Methanoplanus
OTU0172
Methanogenium frigidum (FR749908)
Methanogenium marinum (DQ177344)
Methanogenium cariaci (FR733663)
Methanogenium organophilum (M59131)
Methanofollis
OTU0169
OTU0167
OTU0168
OTU0170
Methanocella arvoryzae MRE50 (AM114193)
Methanocella arvoryzae MRE50 (AM114193)
Methanocella arvoryzae MRE50 (AM114193)
OTU0163
Methanocella paludicola SANAE (AP011532)
Methanocella paludicola SANAE (AP011532)
Methanocella paludicola SANAE (AB196288)
Methanosarcina
ANME−3
ANME−2a/b
ANME−2e (ANME−2c sister clade)
Methanosaeta
F99a103
ANME−2d
Methanoregula
Methanosphaerula
Methanoculleus
Methanogenium
Methanocella
Methanosarcinales
Methanomicrobiales
Methanocellales

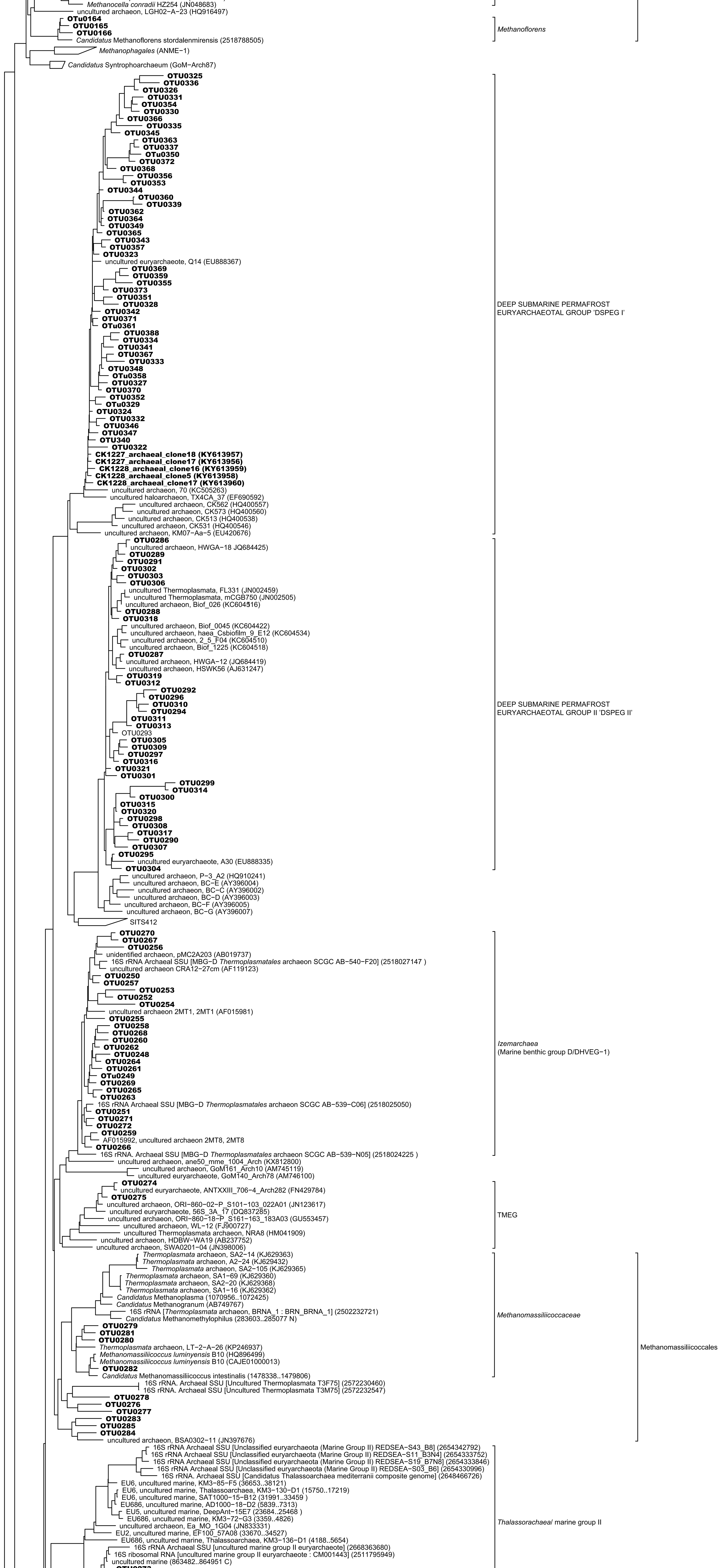

Methanocella conradii HZ254 (JN048683)
uncultured archaeon, LGH02-A-23 (HQ916497)
OTu0164
OTU0165
OTU0166
Candidatus Methanoflorens stordalenmirensis (2518788505)
Methanoflorens
Methanophagales (ANME-1)
Candidatus Syntrophoarchaeum (GoM-Arch87)
OTU0325
OTU0336
OTU0326
OTU0331
OTU0354
OTU0330
OTU0366
OTU0335
OTU0345
OTU0363
OTU0337
OTu0350
OTU0372
OTU0368
OTU0356
OTU0353
OTU0344
OTU0360
OTU0339
OTU0362
OTU0364
OTU0349
OTU0365
OTU0343
OTU0357
OTU0323
uncultured euryarchaeote, Q14 (EU888367)
OTU0369
OTU0359
OTU0355
OTU0373
OTU0351
OTU0328
OTU0342
OTU0371
OTu0361
OTU0388
OTU0334
OTU0341
OTU0367
OTU0333
OTU0348
OTu0358
OTU0327
OTU0370
OTU0352
OTu0329
OTU0324
OTU0332
OTU0346
OTU0347
OTU340
OTU0322
CK1227_archaeal_clone18 (KY613957)
CK1227_archaeal_clone17 (KY613956)
CK1228_archaeal_clone16 (KY613959)
CK1228_archaeal_clone5 (KY613958)
CK1228_archaeal_clone17 (KY613960)
uncultured archaeon, 70 (KC505263)
uncultured haloarchaeon, TX4CA_37 (EF690592)
uncultured archaeon, CK562 (HQ400557)
uncultured archaeon, CK573 (HQ400560)
uncultured archaeon, CK513 (HQ400538)
uncultured archaeon, CK531 (HQ400546)
uncultured archaeon, KM07-Aa-5 (EU420676)
DEEP SUBMARINE PERMAFROST
EURYARCHAEOTAL GROUP 'DSPEG I'
OTU0286
uncultured archaeon, HWGA-18 JQ684425)
OTU0289
OTU0291
OTU0302
OTU0303
OTU0306
uncultured Thermoplasmata, FL331 (JN002459)
uncultured Thermoplasmata, mCGB750 (JN002505)
uncultured archaeon, Biof_026 (KC604516)
OTU0288
OTU0318
uncultured archaeon, Biof_0045 (KC604422)
uncultured archaeon, haea_Csbiofilm_9_E12 (KC604534)
uncultured archaeon, 2_5_F04 (KC604510)
uncultured archaeon, Biof_1225 (KC604518)
OTU0287
uncultured archaeon, HWGA-12 (JQ684419)
uncultured archaeon, HSWK56 (AJ631247)
OTU0319
OTU0312
OTU0292
OTU0296
OTU0310
OTU0294
OTU0311
OTU0313
OTU0293
OTU0305
OTU0309
OTU0297
OTU0316
OTU0321
OTU0301
OTU0299
OTU0314
OTU0300
OTU0315
OTU0320
OTU0298
OTU0308
OTU0317
OTU0290
OTU0307
OTU0295
uncultured euryarchaeote, A30 (EU888335)
OTU0304
DEEP SUBMARINE PERMAFROST
EURYARCHAEOTAL GROUP II 'DSPEG II'
uncultured archaeon, P-3_A2 (HQ910241)
uncultured archaeon, BC-E (AY396004)
uncultured archaeon, BC-C (AY396002)
uncultured archaeon, BC-D (AY396003)
uncultured archaeon, BC-F (AY396005)
uncultured archaeon, BC-G (AY396007)
SITS412
OTU0270
OTU0267
OTU0256
unidentified archaeon, pMC2A203 (AB019737)
16S rRNA Archaeal SSU [MBG-D Thermoplasmatales archaeon SCGC AB-540-F20] (2518027147)
uncultured archaeon CRA12-27cm (AF119123)
OTU0250
OTU0257
OTU0253
OTU0252
OTU0254
uncultured archaeon 2MT1, 2MT1 (AF015981)
OTU0255
OTU0258
OTU0268
OTU0260
OTU0262
OTU0248
OTU0264
OTU0261
OTu0249
OTU0269
OTU0265
OTU0263
16S rRNA Archaeal SSU [MBG-D Thermoplasmatales archaeon SCGC AB-539-C06] (2518025050)
OTU0251
OTU0271
OTU0272
OTU0259
AF015992, uncultured archaeon 2MT8, 2MT8
OTU0266
16S rRNA. Archaeal SSU [MBG-D Thermoplasmatales archaeon SCGC AB-539-N05] (2518024225)
Izemarchaea
(Marine benthic group D/DHVEG-1)
uncultured archaeon, ane50_mme_1004_Arch (KX812800)
uncultured archaeon, GoM161_Arch10 (AM745119)
uncultured euryarchaeote, GoM140_Arch78 (AM746100)
OTU0274
uncultured euryarchaeote, ANTXXIII_706-4_Arch282 (FN429784)
OTU0275
uncultured archaeon, ORI-860-02-P_S101-103_022A01 (JN123617)
uncultured euryarchaeote, 56S_3A_17 (DQ837285)
uncultured archaeon, ORI-860-18-P_S161-163_183A03 (GU553457)
uncultured archaeon, WL-12 (FJ900727)
uncultured Thermoplasmata archaeon, NRA8 (HM041909)
uncultured archaeon, HDBW-WA19 (AB237752)
uncultured archaeon, SWA0201-04 (JN398006)
TMEG
Thermoplasmata archaeon, SA2-14 (KJ629363)
Thermoplasmata archaeon, A2-24 (KJ629432)
Thermoplasmata archaeon, SA2-105 (KJ629365)
Thermoplasmata archaeon, SA1-69 (KJ629360)
Thermoplasmata archaeon, SA2-20 (KJ629368)
Thermoplasmata archaeon, SA1-16 (KJ629362)
Candidatus Methanoplasma (1070956..1072425)
Candidatus Methanogranum (AB749767)
16S rRNA [Thermoplasmata archaeon, BRNA_1 : BRN_BRNA_1] (2502232721)
Candidatus Methanomethylophilus (283603..285077 N)
OTU0279
OTU0281
OTU0280
Thermoplasmata archaeon, LT-2-A-26 (KP246937)
Methanomassiliicoccus luminyensis B10 (HQ896499)
Methanomassiliicoccus luminyensis B10 (CAJE01000013)
OTU0282
Candidatus Methanomassiliicoccus intestinalis (1478338..1479806)
Methanomassiliicoccaceae
16S rRNA. Archaeal SSU [Uncultured Thermoplasmata T3F75] (2572230460)
16S rRNA. Archaeal SSU [Uncultured Thermoplasmata T3M75] (2572232547)
OTU0278
OTU0276
OTU0277
OTU0283
OTU0285
OTU0284
uncultured archaeon, BSA0302-11 (JN397676)
Methanomassiliicoccales
16S rRNA Archaeal SSU [Unclassified euryarchaeota (Marine Group II) REDSEA-S43_B8] (2654342792)
16S rRNA Archaeal SSU [Unclassified euryarchaeota (Marine Group II) REDSEA-S11_B3N4] (2654333752)
16S rRNA Archaeal SSU [Unclassified euryarchaeota (Marine Group II) REDSEA-S19_B7N8] (2654333846)
16S rRNA Archaeal SSU [Unclassified euryarchaeota (Marine Group II) REDSEA-S03_B6] (2654330996)
16S rRNA. Archaeal SSU [Candidatus Thalassoarchaea mediterranii composite genome] (2648466726)
EU6, uncultured marine, KM3-85-F5 (36653..38121)
EU6, uncultured marine, Thalassoarchaea, KM3-130-D1 (15750..17219)
EU6, uncultured marine, SAT1000-15-B12 (31991..33459)
EU686, uncultured marine, AD1000-18-D2 (5839..7313)
EU5, uncultured marine, DeepAnt-15E7 (23684..25468)
EU686, uncultured marine, KM3-72-G3 (3359..4826)
uncultured archaeon, Ea_MO_1G04 (JN833331)
EU2, uncultured marine, EF100_57A08 (33670..34527)
EU686, uncultured marine, Thalassoarchaea, KM3-136-D1 (4188..5654)
Thalassorachaea/ marine group II
16S rRNA Archaeal SSU [uncultured marine group II euryarchaeote] (2668363680)
16S ribosomal RNA [uncultured marine group II euryarchaeote : CM001443] (2511795949)
uncultured marine (863482..864951 C)

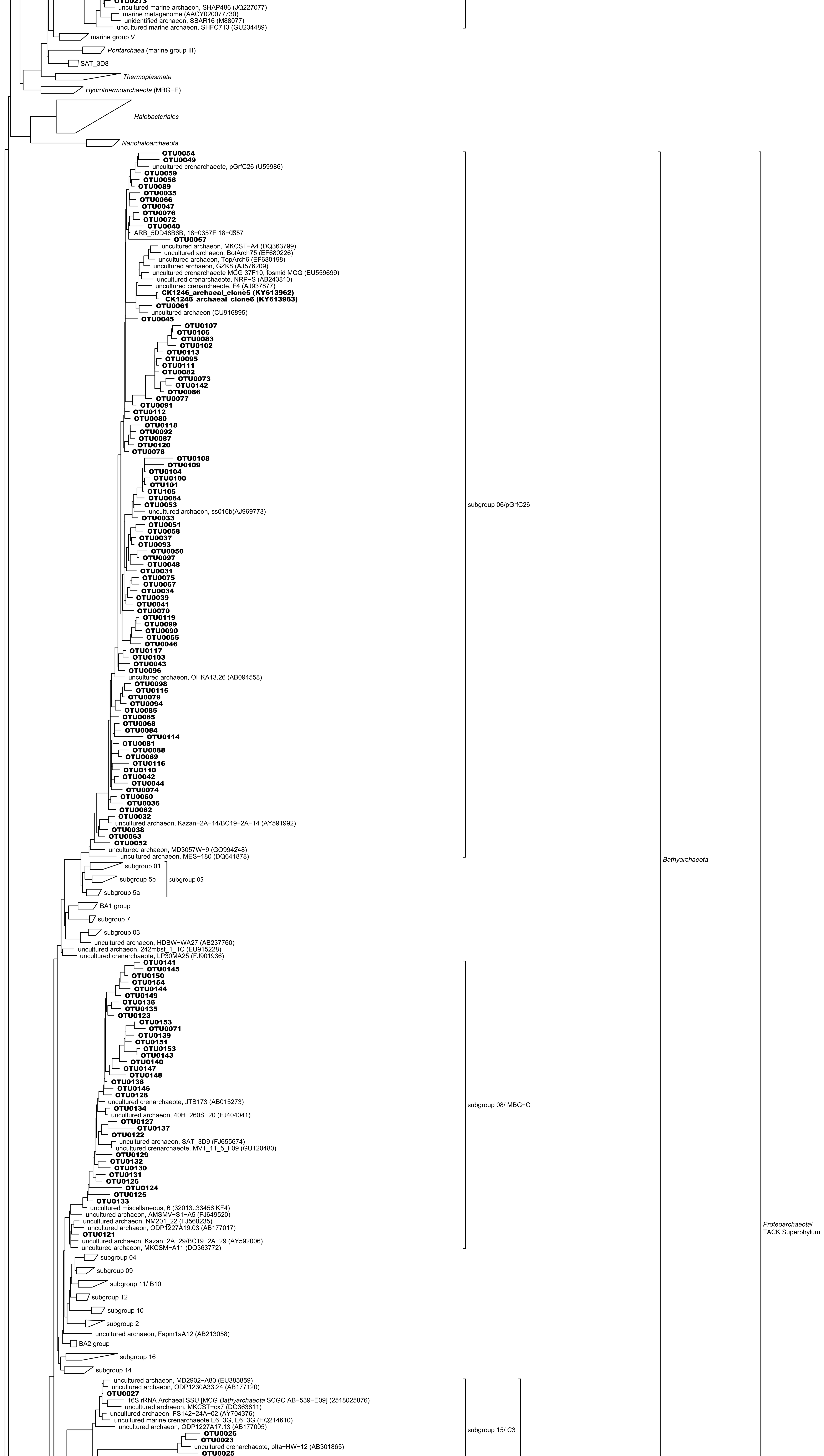
OTU0273
uncultured marine archaeon, SHAP486 (JQ227077)
marine metagenome (AACY020077730)
unidentified archaeon, SBAR16 (M88077)
uncultured marine archaeon, SHFC713 (GU234489)
marine group V
Pontarchaea (marine group III)
SAT_3D8
Thermoplasmata
Hydrothermoarchaeota (MBG-E)
Halobacteriales
Nanohaloarchaeota
OTU0054
OTU0049
uncultured crenarchaeote, pGrfC26 (U59986)
OTU0059
OTU0056
OTU0089
OTU0035
OTU0066
OTU0047
OTU0076
OTU0072
OTU0040
ARB_5DD48B6B, 18-0357F 18-0B57
OTU0057
uncultured archaeon, MKCST-A4 (DQ363799)
uncultured archaeon, BotArch75 (EF680226)
uncultured archaeon, TopArch6 (EF680198)
uncultured archaeon, GZK8 (AJ576209)
uncultured crenarchaeote MCG 37F10, fosmid MCG (EU559699)
uncultured crenarchaeote, NRP-S (AB243810)
uncultured crenarchaeote, F4 (AJ937877)
CK1246_archaeal_clone5 (KY613962)
CK1246_archaeal_clone6 (KY613963)
OTU0061
uncultured archaeon (CU916895)
OTU0045
OTU0107
OTU0106
OTU0083
OTU0102
OTU0113
OTU0095
OTU0111
OTU0082
OTU0073
OTU0142
OTU0086
OTU0077
OTU0091
OTU0112
OTU0080
OTU0118
OTU0092
OTU0087
OTU0120
OTU0078
OTU0108
OTU0109
OTU0104
OTU0100
OTU101
OTU105
OTU0064
OTU0053
uncultured archaeon, ss016b(AJ969773)
OTU0033
OTU0051
OTU0058
OTU0037
OTU0093
OTU0050
OTU0097
OTU0048
OTU0031
OTU0075
OTU0067
OTU0034
OTU0039
OTU0041
OTU0070
OTU0119
OTU0099
OTU0090
OTU0055
OTU0046
OTU0117
OTU0103
OTU0043
OTU0096
uncultured archaeon, OHKA13.26 (AB094558)
OTU0098
OTU0115
OTU0079
OTU0094
OTU0085
OTU0065
OTU0068
OTU0084
OTU0114
OTU0081
OTU0088
OTU0069
OTU0116
OTU0110
OTU0042
OTU0044
OTU0074
OTU0060
OTU0036
OTU0062
OTU0032
uncultured archaeon, Kazan-2A-14/BC19-2A-14 (AY591992)
OTU0038
OTU0063
OTU0052
uncultured archaeon, MD3057W-9 (GQ994248)
uncultured archaeon, MES-180 (DQ641878)
subgroup 06/pGrfC26
subgroup 01
subgroup 5b
subgroup 05
subgroup 5a
BA1 group
subgroup 7
subgroup 03
uncultured archaeon, HDBW-WA27 (AB237760)
uncultured archaeon, 242mbsf_1_1C (EU915228)
uncultured crenarchaeote, LP30MA25 (FJ901936)
OTU0141
OTU0145
OTU0150
OTU0154
OTU0144
OTU0149
OTU0136
OTU0135
OTU0123
OTU0153
OTU0071
OTU0139
OTU0151
OTU0153
OTU0143
OTU0140
OTU0147
OTU0148
OTU0138
OTU0146
OTU0128
uncultured crenarchaeote, JTB173 (AB015273)
OTU0134
uncultured archaeon, 40H-260S-20 (FJ404041)
OTU0127
OTU0137
OTU0122
uncultured archaeon, SAT_3D9 (FJ655674)
uncultured crenarchaeote, MV1_11_5_F09 (GU120480)
OTU0129
OTU0132
OTU0130
OTU0131
OTU0126
OTU0124
OTU0125
OTU0133
uncultured miscellaneous, 6 (32013..33456 KF4)
uncultured archaeon, AMSMV-S1-A5 (FJ649520)
uncultured archaeon, NM201_22 (FJ560235)
uncultured archaeon, ODP1227A19.03 (AB177017)
OTU0121
uncultured archaeon, Kazan-2A-29/BC19-2A-29 (AY592006)
uncultured archaeon, MKCSM-A11 (DQ363772)
subgroup 08/ MBG-C
subgroup 04
subgroup 09
subgroup 11/ B10
subgroup 12
subgroup 10
subgroup 2
uncultured archaeon, Fapm1aA12 (AB213058)
BA2 group
subgroup 16
subgroup 14
Bathyarchaeota
Proteoarchaeota/
TACK Superphylum
uncultured archaeon, MD2902-A80 (EU385859)
uncultured archaeon, ODP1230A33.24 (AB177120)
OTU0027
16S rRNA Archaeal SSU [MCG Bathyarchaeota SCGC AB-539-E09] (2518025876)
uncultured archaeon, MKCST-cx7 (DQ363811)
uncultured archaeon, FS142-24A-02 (AY704376)
uncultured marine crenarchaeote E6-3G, E6-3G (HQ214610)
uncultured archaeon, ODP1227A17.13 (AB177005)
OTU0026
OTU0023
uncultured crenarchaeote, pItta-HW-12 (AB301865)
OTU0025
subgroup 15/ C3

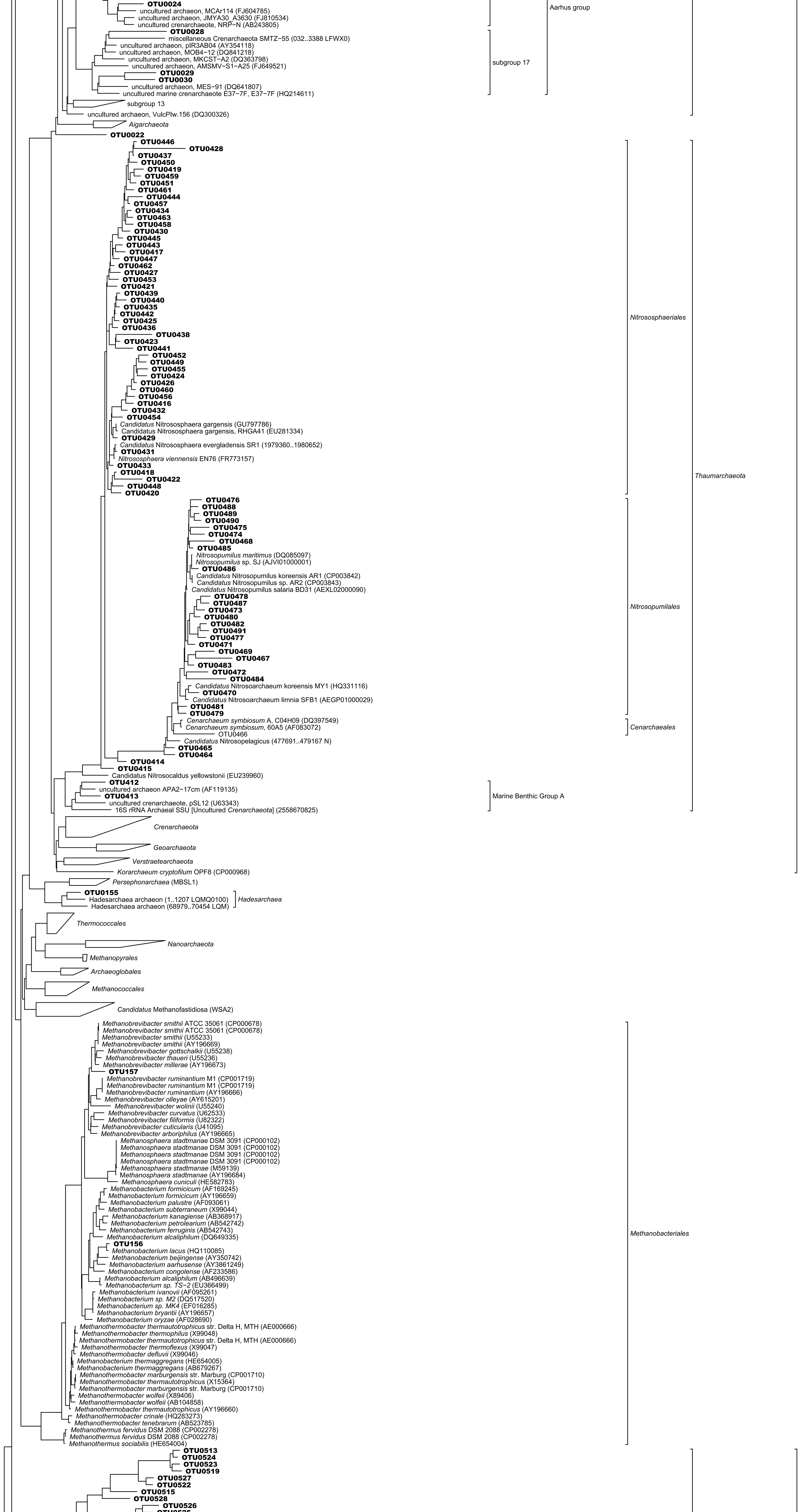
OTU0024
uncultured archaeon, MCAr114 (FJ604785)
uncultured archaeon, JMYA30_A3630 (FJ810534)
uncultured crenarchaeote, NRP−N (AB243805)
Aarhus group
OTU0028
miscellaneous Crenarchaeota SMTZ−55 (032..3388 LFWX0)
uncultured archaeon, pIR3AB04 (AY354118)
uncultured archaeon, MOB4−12 (DQ841218)
uncultured archaeon, MKCST−A2 (DQ363798)
uncultured archaeon, AMSMV−S1−A25 (FJ649521)
subgroup 17
OTU0029
OTU0030
uncultured archaeon, MES−91 (DQ641807)
uncultured marine crenarchaeote E37−7F, E37−7F (HQ214611)
subgroup 13
uncultured archaeon, VulcPlw.156 (DQ300326)
Aigarchaeota
OTU0022
OTU0446
OTU0428
OTU0437
OTU0450
OTU0419
OTU0459
OTU0451
OTU0461
OTU0444
OTU0457
OTU0434
OTU0463
OTU0458
OTU0430
OTU0445
OTU0443
OTU0417
OTU0447
OTU0462
OTU0427
OTU0453
OTU0421
OTU0439
OTU0440
OTU0435
OTU0442
OTU0425
OTU0436
OTU0438
OTU0423
OTU0441
OTU0452
OTU0449
OTU0455
OTU0424
OTU0426
OTU0460
OTU0456
OTU0416
OTU0432
OTU0454
Candidatus Nitrososphaera gargensis (GU797786)
Candidatus Nitrososphaera gargensis, RHGA41 (EU281334)
OTU0429
Candidatus Nitrososphaera evergladensis SR1 (1979360..1980652)
OTU0431
Nitrososphaera viennensis EN76 (FR773157)
OTU0433
OTU0418
OTU0422
OTU0448
OTU0420
Nitrososphaeriales
OTU0476
OTU0488
OTU0489
OTU0490
OTU0475
OTU0474
OTU0468
OTU0485
Nitrosopumilus maritimus (DQ085097)
Nitrosopumilus sp. SJ (AJVI01000001)
OTU0486
Candidatus Nitrosopumilus koreensis AR1 (CP003842)
Candidatus Nitrosopumilus sp. AR2 (CP003843)
Candidatus Nitrosopumilus salaria BD31 (AEXL02000090)
OTU0478
OTU0487
OTU0473
OTU0480
OTU0482
OTU0491
OTU0477
OTU0471
OTU0469
OTU0467
OTU0483
OTU0472
OTU0484
Candidatus Nitrosoarchaeum koreensis MY1 (HQ331116)
OTU0470
Candidatus Nitrosoarchaeum limnia SFB1 (AEGP01000029)
OTU0481
OTU0479
Nitrosopumilales
Cenarchaeum symbiosum A, C04H09 (DQ397549)
Cenarchaeum symbiosum, 60A5 (AF083072)
OTU0466
Cenarchaeales
Candidatus Nitrosopelagicus (477691..479167 N)
OTU0465
OTU0464
OTU0414
OTU0415
Thaumarchaeota
Candidatus Nitrosocaldus yellowstonii (EU239960)
OTU412
uncultured archaeon APA2−17cm (AF119135)
OTU0413
uncultured crenarchaeote, pSL12 (U63343)
16S rRNA Archaeal SSU [Uncultured Crenarchaeota] (2558670825)
Marine Benthic Group A
Crenarchaeota
Geoarchaeota
Verstraetearchaeota
Korarchaeum cryptofilum OPF8 (CP000968)
Persephonarchaea (MBSL1)
OTU0155
Hadesarchaea archaeon (1..1207 LQMQ0100)
Hadesarchaea archaeon (68979..70454 LQM)
Hadesarchaea
Thermococcales
Nanoarchaeota
Methanopyrales
Archaeoglobales
Methanococcales
Candidatus Methanofastidiosa (WSA2)
Methanobrevibacter smithii ATCC 35061 (CP000678)
Methanobrevibacter smithii ATCC 35061 (CP000678)
Methanobrevibacter smithii (U55233)
Methanobrevibacter smithii (AY196669)
Methanobrevibacter gottschalkii (U55238)
Methanobrevibacter thaueri (U55236)
Methanobrevibacter millerae (AY196673)
OTU157
Methanobrevibacter ruminantium M1 (CP001719)
Methanobrevibacter ruminantium M1 (CP001719)
Methanobrevibacter ruminantium (AY196666)
Methanobrevibacter olleyae (AY615201)
Methanobrevibacter wolinii (U55240)
Methanobrevibacter curvatus (U62533)
Methanobrevibacter filiformis (U82322)
Methanobrevibacter cuticularis (U41095)
Methanobrevibacter arboriphilus (AY196665)
Methanosphaera stadtmanae DSM 3091 (CP000102)
Methanosphaera stadtmanae DSM 3091 (CP000102)
Methanosphaera stadtmanae DSM 3091 (CP000102)
Methanosphaera stadtmanae DSM 3091 (CP000102)
Methanosphaera stadtmanae (M59139)
Methanosphaera stadtmanae (AY196684)
Methanosphaera cuniculi (HE582783)
Methanobacterium formicicum (AF169245)
Methanobacterium formicicum (AY196659)
Methanobacterium palustre (AF093061)
Methanobacterium subterraneum (X99044)
Methanobacterium kanagiense (AB368917)
Methanobacterium petrolearium (AB542742)
Methanobacterium ferruginis (AB542743)
Methanobacterium alcaliphilum (DQ649335)
OTU156
Methanobacterium lacus (HQ110085)
Methanobacterium beijingense (AY350742)
Methanobacterium aarhusense (AY3861249)
Methanobacterium congolense (AF233586)
Methanobacterium alcaliphilum (AB496639)
Methanobacterium sp. TS−2 (EU366499)
Methanobacterium ivanovii (AF095261)
Methanobacterium sp. M2 (DQ517520)
Methanobacterium sp. MK4 (EF016285)
Methanobacterium bryantii (AY196657)
Methanobacterium oryzae (AF028690)
Methanobacteriales
Methanothermobacter thermautotrophicus str. Delta H, MTH (AE000666)
Methanothermobacter thermophilus (X99048)
Methanothermobacter thermautotrophicus str. Delta H, MTH (AE000666)
Methanothermobacter thermoflexus (X99047)
Methanothermobacter defluvii (X99046)
Methanobacterium thermaggregans (HE654005)
Methanobacterium thermaggregans (AB679267)
Methanothermobacter marburgensis str. Marburg (CP001710)
Methanothermobacter thermautotrophicus (X15364)
Methanothermobacter marburgensis str. Marburg (CP001710)
Methanothermobacter wolfeii (X89406)
Methanothermobacter wolfeii (AB104858)
Methanothermobacter thermautotrophicus (AY196660)
Methanothermobacter crinale (HQ283273)
Methanothermobacter tenebrarum (AB523785)
Methanothermus fervidus DSM 2088 (CP002278)
Methanothermus fervidus DSM 2088 (CP002278)
Methanothermus sociabilis (HE654004)
OTU0513
OTU0524
OTU0523
OTU0519
OTU0527
OTU0522
OTU0515
OTU0528
OTU0526

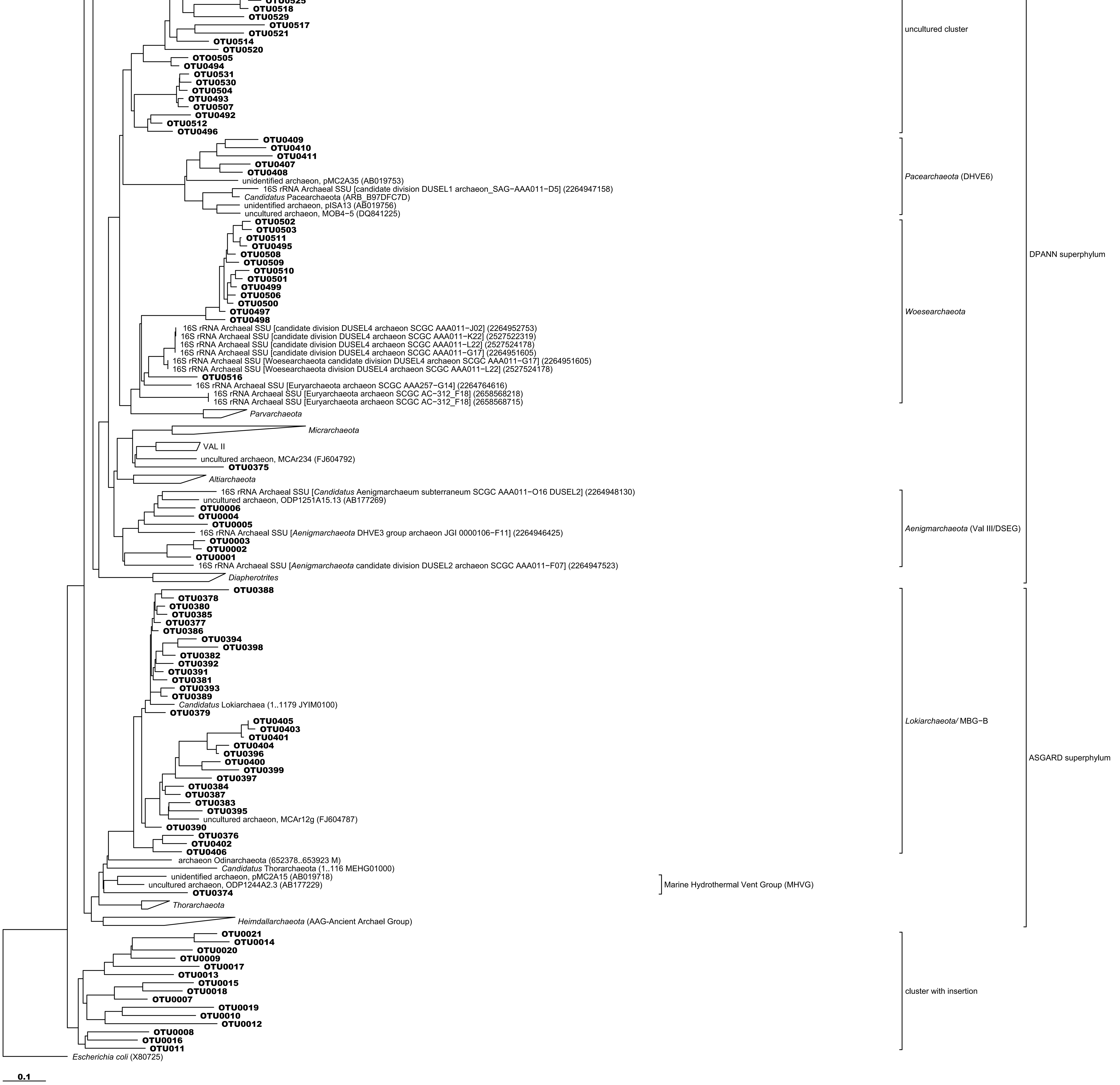

OTU0518
OTU0529
OTU0517
OTU0521
OTU0514
OTU0520
OTU0505
OTU0494
OTU0531
OTU0530
OTU0504
OTU0493
OTU0507
OTU0492
OTU0512
OTU0496
uncultured cluster
OTU0409
OTU0410
OTU0411
OTU0407
OTU0408
unidentified archaeon, pMC2A35 (AB019753)
16S rRNA Archaeal SSU [candidate division DUSEL1 archaeon_SAG−AAA011−D5] (2264947158)
Candidatus Pacearchaeota (ARB_B97DFC7D)
unidentified archaeon, pISA13 (AB019756)
uncultured archaeon, MOB4−5 (DQ841225)
Pacearchaeota (DHVE6)
OTU0502
OTU0503
OTU0511
OTU0495
OTU0508
OTU0509
OTU0510
OTU0501
OTU0499
OTU0506
OTU0500
OTU0497
OTU0498
16S rRNA Archaeal SSU [candidate division DUSEL4 archaeon SCGC AAA011−J02] (2264952753)
16S rRNA Archaeal SSU [candidate division DUSEL4 archaeon SCGC AAA011−K22] (2527522319)
16S rRNA Archaeal SSU [candidate division DUSEL4 archaeon SCGC AAA011−L22] (2527524178)
16S rRNA Archaeal SSU [candidate division DUSEL4 archaeon SCGC AAA011−G17] (2264951605)
16S rRNA Archaeal SSU [Woesearchaeota candidate division DUSEL4 archaeon SCGC AAA011−G17] (2264951605)
16S rRNA Archaeal SSU [Woesearchaeota division DUSEL4 archaeon SCGC AAA011−L22] (2527524178)
OTU0516
16S rRNA Archaeal SSU [Euryarchaeota archaeon SCGC AAA257−G14] (2264764616)
16S rRNA Archaeal SSU [Euryarchaeota archaeon SCGC AC−312_F18] (2658568218)
16S rRNA Archaeal SSU [Euryarchaeota archaeon SCGC AC−312_F18] (2658568715)
Woesearchaeota
Parvarchaeota
Micrarchaeota
VAL II
uncultured archaeon, MCAr234 (FJ604792)
OTU0375
Altiarchaeota
16S rRNA Archaeal SSU [Candidatus Aenigmarchaeum subterraneum SCGC AAA011−O16 DUSEL2] (2264948130)
uncultured archaeon, ODP1251A15.13 (AB177269)
OTU0006
OTU0004
OTU0005
16S rRNA Archaeal SSU [Aenigmarchaeota DHVE3 group archaeon JGI 0000106−F11] (2264946425)
OTU0003
OTU0002
OTU0001
16S rRNA Archaeal SSU [Aenigmarchaeota candidate division DUSEL2 archaeon SCGC AAA011−F07] (2264947523)
Aenigmarchaeota (Val III/DSEG)
Diapherotrites
DPANN superphylum
OTU0388
OTU0378
OTU0380
OTU0385
OTU0377
OTU0386
OTU0394
OTU0398
OTU0382
OTU0392
OTU0391
OTU0381
OTU0393
OTU0389
Candidatus Lokiarchaea (1..1179 JYIM0100)
OTU0379
OTU0405
OTU0403
OTU0401
OTU0404
OTU0396
OTU0400
OTU0399
OTU0397
OTU0384
OTU0387
OTU0383
OTU0395
uncultured archaeon, MCAr12g (FJ604787)
OTU0390
OTU0376
OTU0402
OTU0406
Lokiarchaeota/ MBG−B
archaeon Odinarchaeota (652378..653923 M)
Candidatus Thorarchaeota (1..116 MEHG01000)
unidentified archaeon, pMC2A15 (AB019718)
uncultured archaeon, ODP1244A2.3 (AB177229)
Marine Hydrothermal Vent Group (MHVG)
OTU0374
Thorarchaeota
Heimdallarchaeota (AAG-Ancient Archael Group)
ASGARD superphylum
OTU0021
OTU0014
OTU0020
OTU0009
OTU0017
OTU0013
OTU0015
OTU0018
OTU0007
OTU0019
OTU0010
OTU0012
OTU0008
OTU0016
OTU011
cluster with insertion
Escherichia coli (X80725)
0.1
