# Supplementary material for: Anaerobic methanotrophic communities thrive in deep submarine permafrost
Source: Sci Rep. 2018 Jan 22;8:1291. doi: 10.1038/s41598-018-19505-9 (PMC5778128; doi:10.1038/s41598-018-19505-9)
Supplement: Supplementary file 4 — Supplementary Table S1 [file 41598_2018_19505_MOESM4_ESM.pdf]

Title

Anaerobic methanotrophic communities thrive in deep submarine permafrost

Authors

Matthias Winkel[1,*], Julia Mitzscherling[1], Pier P. Overduin[2], Fabian Horn[1], Maria Winterfeld[3], Ruud Rijkers[1], Mikhail N. Grigoriev[4], Christian Knoblauch[5], Kai Mangelsdorf[6], Dirk Wagner[1], and Susanne Liebner[1]

Affiliations

[1]GFZ German Research Centre for Geosciences, Helmholtz Centre Potsdam, Section 5.3 Geomicrobiology, 14473 Potsdam, Germany

[2]Alfred Wegener Institute, Helmholtz Centre for Polar and Marine Research, Periglacial Research, 14473 Potsdam, Germany

[3]Alfred Wegener Institute, Helmholtz Centre for Polar and Marine Research, Marine Geochemistry, 27570 Bremerhaven, Germany

[4]Mel'nikov Permafrost Institute, SB RAS, Yakutsk, 677010 Russia

[5]Institute of Soil Science, Universität Hamburg, 20146 Hamburg, Germany

[6]GFZ German Research Centre for Geosciences, Helmholtz Centre Potsdam, Section 3.2 Organic Geochemistry, 14473 Potsdam, Germany

*Corresponding author

E-mail: mwinkel@gfz-potsdam.de

Address: Telegrafenberg, building F, room 357, 14473 Potsdam, Germany

					Mamontov Klyk																			Bour Khaya						
					Depth [m bsf]																									
**Phylum/Superclass**	**Class**	**Order**	**Family**	**Genus**	**0,50**	**18,00**	**29,00**	**30,00**	**33,00**	**34,43**	**38,53**	**43,31**	**44,65**	**45,80**	**48,71**	**50,06**	**51,81**	**52,27**	**52,69**	**55,55**	**58,70**	**59,00**	**67,00**	**4,72**	**8,10**	**8,86**	**12,00**	**24.03-24.30**	**24.53-24.68**	**45,46**
**unknown**	-	-	-	-	-	-	-	-	-	-	-	-	-	-	-	0,19	0,37	-	-	-	-	0,06	-	1,12	-	-	-	-	-	-
Aenigmarchaeota	DSEG	-			0,31	-	-	-	-	-	-	-	-	-	-	-	-	-	-	-	-	-	-	-	-	1,42	0,58	-	-	-
Bathyarchaeota	-	-	-	-	-	-	-	-	-	-	-	-	-	-	-	-	-	-	-	-	-	-	-	-	-	-	0,23	-	-	-
	unknown	MCG-6	pGrfC26	-	10,97	52,69	1,55	7,40	5,77	0,79	2,41	6,17	4,23	2,36	78,35	41,28	46,60	55,25	6,42	8,92	17,24	19,86	30,72	10,35	34,23	5,42	6,37	21,29	1,12	3,01
		MCG-8	B10	-	13,31	8,44	0,18	2,83	1,46	-	1,80	5,37	3,26	0,64	0,74	4,06	2,27	1,73	0,98	1,21	2,05	7,62	46,73	27,93	36,57	43,53	42,66	40,58	1,18	2,43
		MCG-15			0,13	-	-	-	-	-	-	-	-	-	-	0,52	-	-	-	-	-	0,38	-	1,90	-	0,14	-	-	-	-
		MCG-17			0,60	-	-	-	-	-	-	-	-	-	-	-	-	-	-	-	-	0,09	-	0,13	-	-	-	-	-	-
Lokiarchaeota	MBG-B/DSAG	-	-	-	14.92	-	-	-	-	-	-	0.58	0.19	0.15	-	0.12	0.35	0.61	-	-	0.32	8.93	0.14	-	4.59	0.63	8.61	-	-	0.18
Euryarchaeota	*Hadesarchaea*	*Hadesarchaeota*	-	SAGMEG-1	-	-	-	-	-	-	-	-	-	-	-	-	-	-	-	-	-	-	-	0,22	-	-	-	-	-	-
	Methanobacteria	*Methanobacteriales*	*Methanobacteriaceae*	*Methanobacterium*	0,18	0,18	0,16	-	-	-	-	0,56	-	-	-	2,99	10,26	-	-	-	0,17	0,50	-	-	0,26	-	-	0,19	0,09	0,08
				Methanobrevibacter	-	-	-	-	-	-	-	-	-	-	-	-	0,12	-	-	-	-	-	-	-	-	-	-	-	-	-
	Methanomicrobia	F99a103	-	-	-	-	-	-	-	-	-	0,61	0,28	-	-	0,27	0,11	-	-	-	0,10	0,09	0,37	-	-	0,08	16,19	-	-	0,13
		Methanocellales	*Methanocellaceae*	*Methanocella*	-	-	-	-	-	-	-	-	-	0,09	-	-	-	-	-	-	-	-	-	-	-	-	-	-	-	-
			Methanoflorentaceae	-	0,13	0,06	4,64	-	1,08	-	0,12	0,44	-	0,68	-	1,13	0,99	-	-	3,30	8,97	1,04	3,30	0,29	0,06	0,09	0,09	-	-	0,12
		Methanomicrobiales	-	-	-	-	0,35	-	0,22	4,70	-	-	-	-	-	0,61	-	0,13	-	-	0,31	0,67	0,22	-	-	-	-	-	-	-
			Methanomicrobiaceae	*Methanoculleus*	-	-	-	-	-	-	-	-	-	-	-	-	0,45	-	-	-	-	-	-	-	-	-	-	-	-	-
				Methanogenium,	-	-	-	-	-	-	-	-	-	0,91	-	0,24	0,36	-	-	0,07	1,39	-	-	-	-	-	-	-	-	-
			Methanoregulaceae		-	-	-	-	-	-	-	-	-	-	-	-	-	-	-	-	-	-	1,25	0,09	-	-	-	-	-	-
				Methanoregula	-	0,07	-	-	-	-	0,06	-	-	0,75	-	5,63	0,43	0,09	-	0,12	0,15	2,78	0,17	0,07	-	0,11	-	7,08	0,09	0,13
				Methanosphaerula	-	-	-	-	-	-	-	-	-	-	0,28	1,55	-	-	-	-	-	-	-	-	-	-	-	-	-	-
		Methanosarcinales	ANME-2a/b	-	-	0,30	-	-	0,60	-	1,23	1,16	-	0,05	0,50	1,12	0,36	-	0,47	0,54	0,59	2,51	0,09	35,86	0,54	33,44	1,21	25,71	19,03	0,85
			ANME-2e		-	0,07	-	-	-	-	-	-	2,35	-	-	-	0,67	-	-	0,12	-	0,16	-	-	15,92	-	-	0,05	0,07	-
			Methanoperedenaceae (ANME-2d)	-	0,84	0,18	0,16	0,70	2,44	1,62	0,38	0,41	6,24	2,67	0,83	0,70	2,86	18,62	70,87	15,97	1,87	3,68	4,35	4,51	2,14	2,44	1,47	0,13	28,29	0,52
			Methanosaetaceae	*Methanosaeta*	0,94	-	-	-	-	-	-	-	-	0,59	-	0,49	0,45	-	-	0,72	-	0,19	1,86	0,07	0,16	0,13	-	-	-	-
			Methanosarcinaceae	ANME-3	-	-	-	-	-	-	-	-	-	-	-	-	0,10	-	1,15	-	-	-	-	-	-	-	-	-	-	-
				Methanosarcina	1,20	0,61	0,22	0,52	1,15	1,05	1,39	9,10	1,80	1,85	1,82	2,81	17,93	18,45	0,20	46,53	6,08	39,14	0,52	0,73	0,65	3,89	5,03	0,51	49,94	35,65
Diaforarchaea	*Izemarchaea*	unknown	MBG-D/DHVEG-1	-	6,11	-	-	-	0,49	-	-	-	0,07	-	-	0,07	0,07	0,08	-	-	-	0,07	0,73	0,06	0,38	0,33	10,08	-	-	-
	Thalassoarchaea	unknown	MG-II	-	2,41	-	-	-	-	-	-	0,06	-	-	-	-	0,08	-	-	-	-	-	-	-	-	-	-	-	-	-
	unknown	unknown	TMEG		-	-	-	-	-	-	-	-	-	-	-	-	-	-	-	-	-	-	-	-	-	-	-	4,45	-	-
		Methanomassiliicoccales	-	-	-	-	-	-	-	-	-	-	-	-	-	-	-	-	-	-	-	-	-	-	0,06	-	0,32	-	-	-
			Methanomassiliicoccaceae	-	-	-	-	-	0,21	-	0,27	-	-	0,28	14,47	0,73	-	-	-	0,38	0,67	0,21	0,69	-	-	0,26	-	-	-	-
	unknown	unknown	ASC21		-	-	-	-	-	-	-	-	-	-	-	-	-	-	-	-	-	-	-	-	-	-	0,25	-	-	-
	unknown	unknown	DSPEG-I	-	2,48	12,19	72,17	83,97	57,46	67,40	77,46	56,31	68,27	20,05	2,62	12,88	9,14	2,26	11,27	19,39	28,14	2,18	5,76	8,62	-	6,88	5,12	-	0,10	44,68
	unknown	unknown	DSPEG-II	-	0,18	-	8,79	0,24	7,51	14,26	2,34	5,60	9,29	3,18	-	0,27	0,25	-	0,07	1,81	5,40	-	0,17	0,28	-	0,06	0,18	-	-	1,18
unknown	unknown	unknown	MEG		-	-	-	-	-	-	-	-	-	-	-	-	0,10	-	-	-	-	-	-	-	-	-	-	-	-	-
Pacearchaeota	*Pacearchaea*	WCHD3-30	-	-	-	-	-	-	-	-	-	-	-	-	-	0,91	-	-	-	-	-	-	-	0,74	-	-	-	-	-	-
Thaumarchaeota	MBG-A	-	-	-	-	-	-	-	-	-	-	-	-	-	-	-	-	-	-	-	-	-	-	-	-	0,24	-	-	-	-
	Nitrosospheria	*Nitrososphaerales*	-	-	0,13	17,48	3,54	0,91	5,26	3,83	11,36	5,15	0,56	8,29	0,29	2,43	0,51	0,14	1,70	0,49	3,29	0,92	0,36	1,52		0,71	0,31	-	0,11	8,47
			Nitrososphaeraceae	-	-	-	-	-	-	-	-	-	-	-	-	-	-	-	-	-	0,11	-	-	-	-	-	-	-	-	-
				Nitrososphaera	1,38	7,74	8,24	3,42	16,37	6,36	0,80	7,71	3,28	57,47	0,09	6,18	1,55	1,58	6,88	0,44	22,65	8,40	2,44	4,90	-	0,21	0,99	-	-	2,56
	unknown	*Cenarchaeales*	*Cenarchaceae*	-	0,08	-	-	-	-	-	-	-	-	-	-	-	-	-	-	-	-	-	-	-	-	-	-	-	-	-
	unknown	*Nitrosopumiluales*	*Nitrosopumilaceae*	*Nitrosopumilus*	43,53	-	-	-	-	-	0,37	0,77	0,20	-	-	-	0,98	1,06	-	-	0,51	0,55	0,15	-	4,46	-	0,31	-	-	-
	unknown	unknown	*Nitrosopelagicus*	-	0,16	-	-	-	-	-	-	-	-	-	-	-	-	-	-	-	-	-	-	-	-	-	-	-	-	-
Woesearchaeota	*Woesearchaea*	YLA114	-	-	-	-	-	-	-	-	-	-	-	-	-	12,84	2,65	-	-	-	-	-	-	0,64	-	-	-	-	-	-
